# Enhancement of colorectal cancer therapy through interruption of the HSF1-HSP90 axis by p53 activation or cell cycle inhibition

**DOI:** 10.1038/s41418-025-01502-x

**Published:** 2025-04-09

**Authors:** Tamara Isermann, Kim Lucia Schneider, Florian Wegwitz, Tiago De Oliveira, Lena-Christin Conradi, Valery Volk, Friedrich Feuerhake, Björn Papke, Sebastian Stintzing, Bettina Mundt, Florian Kühnel, Ute M. Moll, Ramona Schulz-Heddergott

**Affiliations:** 1 Department of Molecular Oncology, University Medical Center Göttingen, Göttingen, Germany; 2 Laboratory of Molecular Tumor Pathology and Systems Biology, Institute of Pathology, Charité – Universitätsmedizin Berlin, Berlin, Germany; 3 German Cancer Consortium (DKTK); Partner Site Berlin, German Cancer Research Center (DKFZ), Heidelberg, Germany; 4 Department of Gynecology and Obstetrics, University Medical Center Göttingen, Göttingen, Germany; 5 Department of General, Visceral, and Pediatric Surgery, University Medical Center Göttingen, Göttingen, Germany; 6 Institute for Pathology, Hannover Medical School, Hannover, Germany; 7 Department of Hematology, Oncology and Cancer Immunology, Charité – Universitätsmedizin Berlin, Berlin, Germany; 8 Department of Gastroenterology, Hepatology, Infectious Diseases and Endocrinology, Hannover Medical School, Hannover, Germany; 9 Department of Pathology, Stony Brook University, Stony Brook, NY USA

**Keywords:** Oncogenes, Tumour-suppressor proteins

## Abstract

The stress-associated chaperone system is an actionable target in cancer therapies. It is ubiquitously upregulated in cancer tissues and enables tumorigenicity by stabilizing oncoproteins. Most inhibitors target the key component, heat-shock protein 90 (HSP90). Although HSP90 inhibitors are highly tumor-selective, they fail in clinical trials. These failures are partly due to interference with a negative regulatory feedback loop in the heat-shock response (HSR): in response to HSP90 inhibition, there is compensatory synthesis of stress-inducible chaperones, mediated by the transcription factor heat-shock-factor 1 (HSF1). We recently identified that wild-type p53 reduces the HSR by repressing HSF1 via a p21-CDK4/6-MAPK-HSF1 axis. Here, we test whether in HSP90-based therapies, simultaneous p53 activation or direct cell cycle inhibition interrupts the deleterious HSF1-HSR axis and improves the efficiency of HSP90 inhibitors. We found that the clinically relevant p53 activator Idasanutlin suppresses the HSF1-HSR activity in HSP90 inhibitor-based therapies. This combination synergistically reduces cell viability and accelerates cell death in p53-proficient colorectal cancer (CRC) cells, murine tumor-derived organoids, and patient-derived organoids (PDOs). Mechanistically, upon combination therapy, CRC cells upregulate p53-associated pathways, apoptosis, and inflammatory pathways. Likewise, in a CRC mouse model, dual HSF1-HSP90 inhibition represses tumor growth and remodels immune cell composition. Importantly, inhibition of the cyclin-dependent kinases 4/6 (CDK4/6) under HSP90 inhibition phenocopies synergistic repression of the HSR in p53-proficient CRC cells. Moreover, in p53-deficient CRC cells, HSP90 inhibition in combination with CDK4/6 inhibitors similarly suppresses the HSF1-HSR and reduces cancer growth. Likewise, p53-mutated PDOs respond to dual HSF1-HSP90 inhibition, providing a strategy to target CRC independent of the p53 status. In sum, we provide new options to improve HSP90-based therapies to enhance CRC therapies.

## Introduction

Colorectal cancer (CRC) is the second most common cause of cancer-related deaths in industrialized countries, leading to nearly 1 million deaths per year [1]. In 2020, CRC was estimated to count for 10% of all new cancer diagnoses and 9.4% of all cancer deaths [2]. Advanced and metastasized CRC stages have a 5-year survival rate of approximately 15% [Morris ASCO]. Thus, new therapeutic strategies are urgently needed, especially for advanced stages.

A promising target in cancer therapy is the chaperone system. During oncogenesis, the normal chaperone function (aiding other proteins in proper folding) is dramatically subverted to enable the malignant lifestyle of tumor cells [3–11]. Consequently, cancer cells are strongly addicted to a ubiquitously upregulated chaperone system [8, 12–14]. The main reason is that cancer cells are under constant and cumulative proteotoxic stress, driven by most hallmarks of cancer [15]. They defend against these pathophysiological stresses by constitutive activation of heat-shock factor 1 (HSF1), the master transcription factor of stress-induced chaperones [16–18]. Thus, HSF1 hyperactivation leads to massive upregulation of stress-inducible heat-shock proteins (HSPs), foremost HSP90α, plus HSP70, other HSPs, and numerous pro-tumorigenic co-chaperones. This cumulates in the formation of tumor cell-specific super-chaperone complexes with HSP90 as a key component. Consequently, HSP90 stabilizes hundreds of mutated and truncated oncoproteins which normally would be degraded by proteasomes. HSP90-stabilized oncogenic proteins, collectively called HSP90 clients, include missense p53 mutants [11, 19–22], receptor tyrosine kinases (ErbB1, ErbB2/HER2) [16, 23], signaling kinases (AKT) [24], hormone receptors [25–27] and cytokines [16, 28].

Since cancer cells are addicted to the HSF1-regulated chaperone system, it represents an attractive tumor-selective therapeutic target. Most of the promising small molecule inhibitors were generated against the N-terminal ATPase domain of HSP90 [6, 7, 29–31]. However, despite encouraging pre-clinical results, most, if not all, clinical oncology trials using HSP90 inhibitors alone or in combination with chemotherapeutics have failed so far [6, 7, 29, 31]. We and others hypothesize that a major reason for the failure might be the compensatory stimulation of HSF1 by the inadvertent interference with the negative regulatory HSP90-HSF1 feedback loop upon HSP90 inhibition. This severely limits the therapeutic efficacy of HSP90 inhibitors. In normal cells, this feedback via inhibitory HSP90-HSF1 protein-protein binding is needed to properly control HSF1 activity. Normally, upon activation, HSF1 trimerizes and binds to its cognate DNA element to induce stress-inducible HSPs such as HSP90α, HSP70s, HSP40s, and co-chaperones – a reaction known as the heat-shock response (HSR) [3–10]. However, to limit and control HSF1 activation and the HSR, the induced target protein HSP90 - through its N-terminal domain - binds back and inactivates HSF1, thus establishing a negative feedback loop. In contrast, upon proteotoxic stress, HSP90 dissociates from HSF1 to re-activate the HSR. Since cancer cells are under constant proteotoxic stress, their negative feedback loop is already partially diminished and thus, HSF1 is hyperactivated as a chronic condition. Pharmacological HSP90 inhibition phenocopies HSP90 disappearance and thus promotes further activation and binding of HSF1 to its cognate DNA response element, thereby fully inducing the unwanted HSR in cancer cells. As a result, tumor-driving proteins might be further stabilized by alternative chaperones other than HSP90 [32–35]. Thus, preventing the deleterious HSR upon HSP90 inhibition in cancer cells is predicted to improve the therapeutic efficacy of clinically relevant HSP90 inhibitors.

Our previous findings might provide a viable approach towards this goal. We recently showed that wild-type p53 (here termed ‘p53’) suppresses HSF1 activity in human CRC cells, in mouse and human CRC-derived organoids, and in a CRC mouse model in vivo [36]. p53, a key tumor suppressor preventing tumor initiation and progression, is activated by a broad spectrum of stresses such as DNA damage, oncogene hyperactivation, hypoxia, or metabolic stress [20, 37, 38]. In response, p53 transcriptionally induces genes to maintain cell integrity through, e.g., transient cell cycle arrest, activation of DNA repair, and if cells are irreparably damaged, induction of cell death. We found that activated p53 suppresses HSF1 activity and its target genes (the HSR) via a downstream CDKN1A/p21-CDK4/6 - MAPK axis [36]. Specifically, the p53-p21 axis induces a cell cycle arrest via CDK4/6 inhibition. Based on these findings, we asked whether HSF1 suppression by either p53 activation or CDK4/6 inhibition prevents the HSR in the context of HSP90 inhibition, thereby allowing a far more profound suppression of chaperone activity. Indeed, we find that p53 activation or CDK4/6 inhibition strongly antagonizes the unwanted activation of HSF1 and HSR upon treatment with HSP90 inhibitors. In the context of HSP90 inhibition, we show here that dual pathway inhibition via p53 activation by RG-7388 (Idasanutlin, a clinically relevant p53 activator) prevents upregulation of HSF1 target gene expression in human CRC cells. Importantly, combination treatment synergistically reduces cell viability and increases apoptosis in CRC cell lines, murine CRC-derived organoids, and patient-derived organoids (PDOs). Notably, in combination therapy, inflammatory pathways are strongly upregulated, indicated by the deregulation of immune cell composition in our CRC mouse model in vivo. Dual pathway inhibition profoundly diminishes tumor sizes without obvious toxicities for mice. Likewise, in p53-deficient CRC cells and PDOs, CDK4/6 inhibition by Palbociclib phenocopies p53 activation and lowers the HSR by repressing HSF1 target genes, causing reduced cancer cell growth.

In sum, our results show that p53 activation or CDK4/6 inhibition can each prevent the HSR rebound response induced by HSP90 inhibition in tumor cells. Dual inhibition improves HSP90-based therapies by (i) abrogating the HSR under HSP90 inhibition, (ii) more efficient degradation of HSP90 clients, and (iii) additional positive effects from the p53 tumor-suppressive program or from cell cycle arrest. This improves the therapeutic effect of the promising class of highly tumor-selective HSP90 inhibitors, independent of the p53 status and independent of the first-line CRC therapy status.

## Methods

All methods were performed in accordance with the relevant guidelines and regulations.

### Mouse experiments

Animal experiments were approved by the Göttingen University Medical Center Ethik Kommission and by the Niedersächsisches Landesamt für Verbraucherschutz und Lebensmittelsicherheit, LAVES, Lower Saxony, Germany. 10-week-old male C57BL/6J mice weighing at least 20 g were used for experiments. The experiments were performed under pathogen-free barrier conditions. To induce colorectal carcinoma (CRC), a single dose of Azoxymethane (AOM, 10 mg/kg in 0.9% sodium chloride, Sigma) was injected intraperitoneally. One-week post AOM injection, acute colitis was induced through the addition of 2% dextran sodium sulfate (DSS, MP Biomedicals) to the drinking water for 6 days. For generating mutp53-harboring murine organoids, mice containing two humanized *TP53*^R248Q^ alleles [36, 39] were treated with 10 mg/kg AOM and 1.5% DSS for 6 days to induce CRC tumors.

5 weeks after AOM induction, tumor growth was evaluated using colonoscopy (Karl Storz GmbH). The Becker & Neurath score was used for tumor size scoring [40]: S1 = just detectable, S2 = 1/8 of the lumen, S3 = 1/4 of the lumen, S4 = 1/2 of the lumen, and S5 > 1/2 of the lumen. Regarding the guidelines and regulations, tumor sizes and scoring were permitted by the ethics commissions. Thereby, the maximal tumor size in animal experiments is scoring S4. Thereby, if several tumors are located on the same colonic lumen area, the total tumor load of this area dictates the score. The maximum tumor size/burden was not exceeded in all experiments.

Once mice had developed at least one S2 tumor and three S1 tumors distributed along the complete colon, treatment commenced. Mice received 50 mg/kg RG-7388 (MedChem Tronica) orally (dissolved in 10% DMSO in 0.5% HMPC/1% Tween 80) 5× per week, 50 mg/kg Ganetespib (Synta Pharmaceuticals) intravenously (dissolved in 10% DMSO/18% Cremophor RH40/3.6% Dextrose in H_2_O) 2× per week, or their combination for the duration of 3 weeks. Using weekly colonoscopies, tumor growth was visualized to follow differences in the treatment.

After 3 weeks of treatment, all mice were euthanized. Colons were harvested, opened longitudinally, and cleaned. Using a caliper, tumor sizes were measured, and tumor numbers were recorded. Where possible, tumor biopsies were taken. Colons were rolled up into “swiss rolls” and fixed in 4% paraformaldehyde/PBS and bisected. For histological processing, both halves were placed into a single cassette and embedded in paraffin.

### Preparation and cultivation of murine small intestinal and colonic tumor organoids

Small intestinal and colonic tumor organoids were prepared as described by Klemke et al. [41] and explained in detail in the supplementary information.

### Cultivation of patient-derived colonic organoids

Clinical samples for preparation of patient-derived organoids (PDO #1 - PDO #3) were generated by the Department of General, Visceral, and Pediatric Surgery of the University Medical Center Göttingen (UMG, Germany) with Approval # 9/8/08 and # 25/3/17 from the UMG Ethics Committee. Tumor PDOs were cultivated in advanced DMEM F-12, supplemented with GlutaMAX, HEPES, Penicillin/Streptomycin, 50% Wnt3a conditioned medium, 20% Rspondin-1 conditioned medium, 10% Noggin conditioned medium, B27, N2, 1.25 mM N-Acetyl-L-Cysteine, 500 nM A-83-01, 10 µM SB202190, 50 ng/mL hEGF, 1 mM nicotinamide and primocin. Normal mucosal PDOs were cultured in an IntestiCult organoid growth medium. During treatment, SB202190 and primocin were removed from the organoid medium.

For PDO #4, a clinical sample was obtained at the Charité Universitätsmedizin Berlin (approval EA1/069/113). The matched pair of PDO #5 was obtained by the Department of Hematology, Oncology, and Cancer Immunology at the Charité Universitätsmedizin Berlin (approval # EA1/161/21). PDOs were cultivated in advanced DMEM F-12, supplemented with GlutaMAX, HEPES, Penicillin/Streptomycin, B27, N2, 1.25 mM N-Acetyl-L-Cysteine, 500 nM A-83-01, 3 µM SB202190, 50 ng/mL hEGF, 20 ng/mL hFGF, 10 mM nicotinamide, and primocin.

Large, multi-volume tumor PDO cultures and normal mucosal PDOs were achieved by using only mechanical disruption during the passaging procedure. Single cell-dissociated tumor PDOs were dissociated during passaging by incubation in Trypsin/Rho-kinase Inhibitor/DNase solution for 15 min, followed by mechanical disruption.

### Multiplex immunohistochemistry (mIHC)

mIHC staining was performed according to the manufacturer’s instructions (Opal^TM^ 4-Color Anti-Rabbit Automation IHC; Opal^TM^ Polymer Anti-Rabbit HRP Secondary Antibody Kit; Akoya Biosciences^®^). The following primary rabbit antibodies were used for staining: CD4 (CST25229; 1:200), CD8 (CST98941; 1:400), F4/80 (CST 70076; 1:1000), and Ly6G (CST 87048; 1:1000) were purchased from Cell Signaling Technology^®^; CD11b (Ab133357; 1:3000) from Abcam^®^; PanCytokeratin (NBP3-07280; 1:1000) from Novus Biologicals^®^. Nuclei were stained with DAPI (Akoya Biosciences^®^). Slides were scanned using the “Vectra Polaris” platform (Akoya Biosciences^®^). Spectral unmixing and further tissue/cell segmentation, as well as phenotyping, were performed using the inForm Advanced Image Analysis software (inForm v2.4.10, Akoya Biosciences^®^). Multiplex data analyses: Data output from inForm 2.4.10 was further processed in RStudio (Rstudio v1.1.456) using “R” packages “Phenoptr” and “PhenoptrReports” with R version 4.1.0. Consolidated results for “Cell densities” were exported and used for further analyses.

### Cell viability in human cancer cells

Using CellTiter-Glo^®^ Luminescent Cell Viability Assay (Promega), cell viability was assessed according to the manufacturer’s guidelines. Biological replicates were measured in triplicates and viability was normalized to DMSO control. HSA (Highest Single Agent) synergy scores were calculated using the synergyfinder.fimm.fi (version 3.0) web application. Synergy scores: <−10 antagonistic, −10 to 10 additive, and >10 synergistic.

The definition of the HSA score (or Gaddum’s non-interaction model) is: “It assumes that the expected combination effect equals to the higher individual drug effect at the dose in the combination, representing the idea that a synergistic drug combination should produce additional benefits on top of what its components can achieve alone” [42, 43].

### mRNA sequencing of HCT116 cells

HCT116 cells were treated for 24 h with DMSO, 50 nM Ganetespib, 1 µM RG-7388, or in combination. RNA samples were generated as pools. In detail, two biological replicates, each consisting of two pooled biological replicates per treatment group (total *n* = 4 per group; DMSO, Ganet, RG-7388, combi), were analyzed. RNA samples were sequenced by Novogene (Cambridge Science), including mRNA library preparation (poly A enrichment), NovaSeq 6000 PE150 sequencing, and raw Data quality control. Raw sequencing data were then processed in the high-performance computing cluster provided by the Gesellschaft für wissenschaftliche Datenverarbeitung mbH Göttingen (https://www.gwdg.de/). Quality check was performed with FastQC (version 0.11.4). Reads were trimmed (11 bp from the 5′ end), FASTQ Trimmer (FASTX toolkit version 0.0.14), aligned to the human genome (GRCh38.106), and assigned to genomic features with RNA STAR (version 2.7). DESeq2 (version 2.11.40.6) was carried out in the GALAXY environment (https://galaxy.gwdg.de) with default parameters for differential expression analyses. For WTp53, target genes normalized counts generated by DESeq2 were used to perform GSEA analysis (Broad Institute, version 4.1.0) with the following parameters: number of permutations = 1000, type = gene_set, no_collapse, max size = 1000, and min size = 15. For HSF1 analysis, the HSF1 target gene set was extracted from [44] (Supplementary Data, upregulated genes after heat-shocked HeLa cells) and uploaded into GSEA as a GMX file.

### Quantification, statistical analysis, and original immunoblots

Statistics of each experiment (number of animals, number of tumors, biological replicates, technical replicates, and precision measures (mean and ±SEM)) are provided in the figures and figure legends. For levels of significance, the following designations were used within this manuscript: *p ≤ 0.05; **p ≤ 0.01; ***p ≤ 0.001; ns not significant.

Full and uncropped western blots/immunoblots are presented as Original Data Files.

Further methods are provided as Supplementary Methods within the Supplementary Information.

## Results

### Dual HSF1-HSP90 inhibition via p53 activation synergistically impairs colorectal cancer cell growth by abrogating the heat-shock response

HSP90 inhibitors (HSP90i) are highly tumor-selective and represent a promising class of anti-cancer drugs [3–11, 16, 20, 28, 45–48]. Although HSP90 inhibition alone shows strong anti-tumorigenic effects in experimental mouse models in vivo, most HSP90 inhibitors have so far failed in clinical trials, one reason being the rebound HSF1-induced heat-shock response (HSR) upon HSP90 inhibition [5, 10, 13, 29]. Since we recently demonstrated that the HSF1-governed HSR is suppressed by wild-type p53 (p53) activation upon Nutlin-3a [36], we tested the strategy of counter-regulating the unwanted HSR through HSF1 suppression by p53 activation in p53-proficient tumors within HSP90-based therapies. Approx. 40% of human CRC tumors harbor wild-type p53, thus presenting a significant proportion of colorectal cancer (CRC) patients.

To activate p53 we used the clinically advanced Nutlin-3a derivate Idasanutlin (RG-7388), an MDM2 inhibitor currently in numerous clinical trials. MDM2 is the principal antagonist of p53. Idasanutlin disrupts p53’s interaction with MDM2 and thus blocks its E3 ligase-mediated proteasomal degradation, thereby stabilizing and activating p53 [38, 49, 50]. We chose this highly specific non-genotoxic mode of p53 activation to avoid generating confounding DNA damage with its multiple activated pathways that can contribute to mutually contradictory HSF1 modifications [51, 52]. For HSP90 inhibition we used Ganetespib and Onalespib, both currently also in clinical trials.

**Fig. 1:**
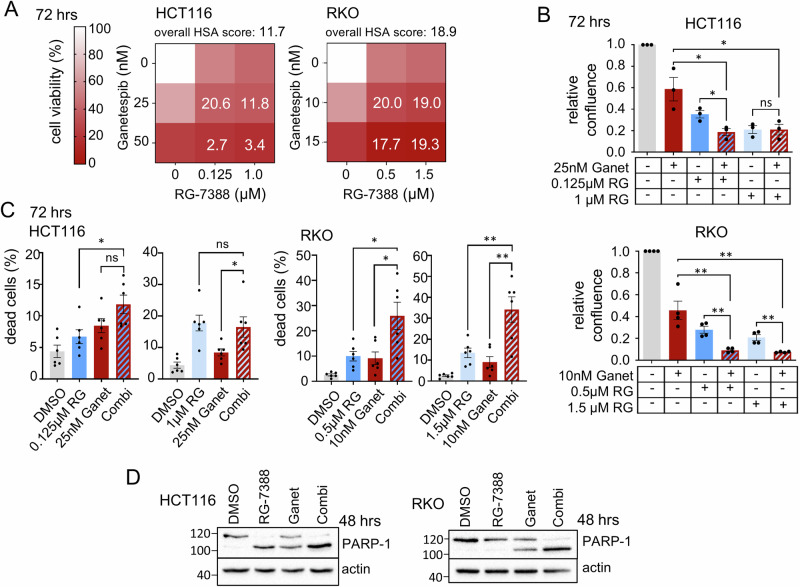
Dual HSP90-HSF1 inhibition via p53 activation synergistically impairs colorectal cancer cell growth. **A** Cell viability matrices of HCT116 (left) and RKO (right) cells treated with Ganetespib – RG-7388 combinations for 72 h at indicated concentrations. Color scheme represents changes in cell viability. Numbers within the matrix indicate the HSA synergy score. Synergy scores: <−10 antagonistic; −10 to 10 additive; >10 synergistic. ≥3 biological replicates. **B** Relative confluence after 72 h treatment of HCT116 (top) and RKO (bottom) cells. Cell confluence was analyzed by Celigo imaging cytometer. Confluence relative to DMSO control, set at value 1. **C** Induction of cell death. PI/Hoechst/Annexin V staining of HCT116 (left) and RKO (right) cells treated for 72 h with Ganetespib and RG-7388 at the indicated concentrations. Percent dead cells include PI+ only, annexin V+ only and PI+Annexin V+ cells and were analyzed by Celigo imaging cytometer. **D** PARP-1 immunoblots of HCT116 (left) and RKO (right) cells treated for 48 h. HCT116 were treated with 50 nM Ganet and 1 μM RG. RKO were treated with 25 nM Ganet and 1.5 μM RG. Representative immunoblots shown from 3 replicates with 2 independent experiments each. For HCT116 cells, actin corresponding to PARP-1 is in parallel shown in Fig. 2D for tAKT staining. PARP-1 and tAKT were processed in parallel on the same membrane, and consequently actin as loading control is shown twice. **B**, **C** Mean ± SEM from ≥3 biological replicates, Student’s *t*-test, *p ≤ 0.05, **p ≤ 0.01, ***p ≤ 0.001; ns not significant. Ganet: Ganetespib, RG: RG-7388.

Initially, we identified effective therapeutic concentrations for single drugs in cell viability assays of CRC cell lines (Fig. S1A, B). We chose two different concentrations with ICs of 80%–70% and approx. 50% of single drugs for combination treatments of the respective single drugs. Tumor cell growth and morphological changes were also monitored upon drug responses. As shown in Figs. 1A and S1C, combination treatment of Ganetespib plus RG-7388 yielded additive to synergistic reduction of cell viability in p53-proficient CRC cell lines HCT116 and RKO, compared to single treatments. Combination treatments showed stronger cell viability effects for all tested drug concentrations compared to corresponding single drugs of the same concentration (Figs. 1A and S1C). Of note, synergistic effects were p53-dependent. In contrast to p53-proficient cells, isogenic HCT116 cells harboring a homozygous p53 deletion failed to show stronger effects upon dual HSF1-HSP90 pathway inhibition (Fig. S1D).

Similarly, cancer cell confluence decreased further upon p53 activation with combinatorial HSP90i plus RG-7388 treatment compared to single treatments (Figs. 1B and S1E). Likewise, combination treatments induced stronger cell death at different drug concentrations compared to single treatments (Fig. 1C). Although cell death was only modest in HCT116 cells (Fig. 1C), PARP cleavage was readily detectable in both cell lines (Fig. 1D). In line, a shorter 48 h dual pathway inhibition also increased cell death in RKO cells, but not in HCT116 cells compared to single treatment (Fig. S1F). p53 activation by RG-7388 also diminished cell viability upon combinatorial Onalespib treatment compared to single treatments (Fig. S1G), although the effects were milder than for Ganetespib (compare to Fig. S1C).

**Fig. 2:**
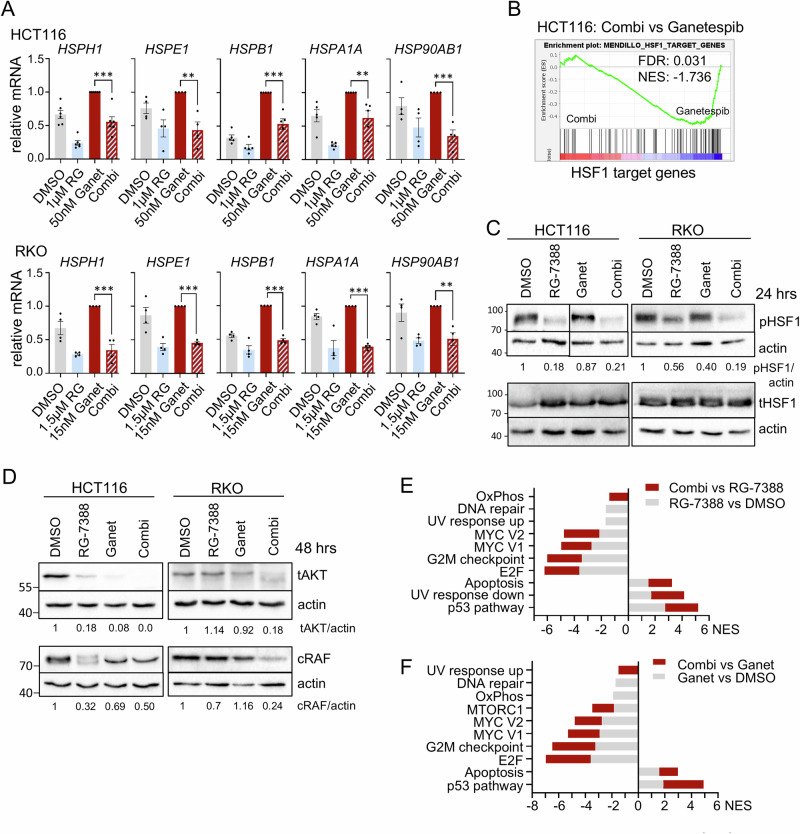
Dual HSP90-HSF1 inhibition via p53 activation abrogates the HSF1-mediated HSR and destabilizes HSP90 clients. **A** mRNA expression levels of HSF1 target genes in HCT116 (top) and RKO (bottom) cells treated for 24 h. qRT-PCRs for the indicated mRNAs normalized to *RPLP0* mRNA. Mean ± SEM from ≥ 3 biological replicates. Student’s *t*-test, *p ≤ 0.05, **p ≤ 0.01, ***p ≤ 0.001; ns not significant. **B** GSEA enrichment plot for HSF1 target genes in HCT116 cells treated with drug combination (1 µM RG-7388 plus 50 nM Ganetespib) versus Ganetespib alone (50 nM). HSF1 target gene list from [72]. **C**, **D** Immunoblots for HCT116 and RKO cells treated with 1 µM RG-7388, 50 nM Ganetespib for HCT116, or 1.5 µM RG-7388, 15 nM Ganetespib for RKO cells for 24 h (**C**) or 48 h (**D**). **C** Blots were probed for phospho-Ser326 HSF1 (pHSF1) and total HSF1 (tHSF1). For RKO cells, actin corresponding to pHSF1 is in parallel shown in Fig. S2D for p53 staining, because pHSF1 and p53 were processed in parallel on the same membrane, and consequently actin as loading control is shown twice. **D** Blots were probed for total AKT (tAKT) and cRAF. Densitometric expression levels normalized for actin are indicated. For HCT116 cells, actin corresponding to tAKT is in parallel shown in Fig. 1D for PARP-1 staining. tAKT and PARP-1 were processed in parallel on the same membrane, and consequently actin as loading control is shown twice. **E**, **F** GSEA pathway analysis for hallmark gene sets on RNAseq data from HCT116 cells treated for 24 h with DMSO, 50 nM Ganetespib, 1 µM RG-7388 or in combination as in (**B**) comparing drug combination versus RG-7388 alone (**E**) or Ganetespib alone (**F**). Gray bars, NES (Normalized Enrichment Score) of RG-7388 (**E**) or Ganetespib (**F**) treatment relative to DMSO. Red bars, NES of combination treatment relative to either RG-7388 or Ganetespib treatment alone, respectively.

Since we hypothesized that the observed synergistic effects arise from the abrogation of the HSP90-HSF1 negative feedback loop, we analyzed the heat-shock response (HSR) upon concomitant p53 activation in HSP90-based therapy by quantitative real-time PCR. Indeed, we found that dual HSF1-HSP90 pathway inhibition suppresses the increase of HSF1 target gene expression compared to Ganetespib alone in HCT116 and RKO cells, using a range of drug concentrations (Figs. 2A and S2A). Randomly selected classical HSF1 targets, including *HSPH1*, *HSPE1*, *HSPB1*, *HSPA1A,* and *HSP90AB1,* were downregulated by concomitant p53 activation during HSP90 inhibition. While not every HSF1 target gene is upregulated upon Ganetespib alone (Figs. 2A and S2A, HSR response, compare gray with red bars), dual HSF1-HSP90 pathway inhibition largely repressed them below the level of untreated DMSO control cells, reflecting a diminished basal HSF1 activity. Even more importantly, RNAseq analysis exhibited that p53 activation combined with HSP90 inhibition reduces global HSF1 target gene expression, compared to the HSR-inducing Ganetespib single treatment (Fig. 2B). Moreover, RNAseq analysis also demonstrated an increase of selected p53 target genes (Fig. S2B) and enrichment plots (Fig. S2C) upon RG-7388 treatment. Western blot analyses at selected RG-7388 doses functionally confirmed p53 protein accumulation, indicating p53 activation under HSP90 inhibition (Fig. S2D). The critical phosphorylation site for HSF1 activation is on Ser326 (pHSF1), which serves as a functional hallmark for the tumor-promoting HSR [12, 16]. Consistent with the observed HSF1 target gene repression, RG-7388 in combination with Ganetespib strongly reduced pSer326-HSF1 levels in HCT116 and RKO cells compared to HSP90 inhibition alone (Fig. 2C). Moreover, RG-7388 combined with Ganetespib destabilizes classical HSP90 clients such as AKT and cRAF more strongly than Ganetespib alone (Fig. 2D), confirming the pronounced inactivation of the HSF1-HSR response and consequently the profound destabilization of HSP90 clients. To further understand the consequences of a dual HSF1-HSP90 pathway repression, RNAseq data were subjected to GSEA pathway analysis (Fig. 2E, F). Indeed, compared to RG-7388 treatment alone (gray bars), RG-7388 in combination with Ganetespib broadly enhanced the hallmarks of p53-associated pathways (Fig. 2E, red bar). The same is seen when comparing the combination versus Ganetespib alone (Fig. 2F, red bar). Conversely, compared to RG-7388 or Ganetespib alone, combination treatment impaired cell cycle-driving pathways, including E2F/RB and DREAM/MuvB target genes, MYC pathways, and the G2M checkpoint more strongly (Fig. 2E, F).

In sum, we conclude that p53 activation effectively suppresses the HSF1-mediated HSR and synergistically impairs human CRC cell growth, thereby improving HSP90-based therapies. Moreover, the powerful tumor-suppressive p53 program can be profoundly activated in the presence of concomitant HSP90 inhibition.

### Activated p53 represses the heat-shock response in HSP90-based therapies in murine colorectal tumor-derived organoids and CRC patient-derived organoids

**Fig. 3:**
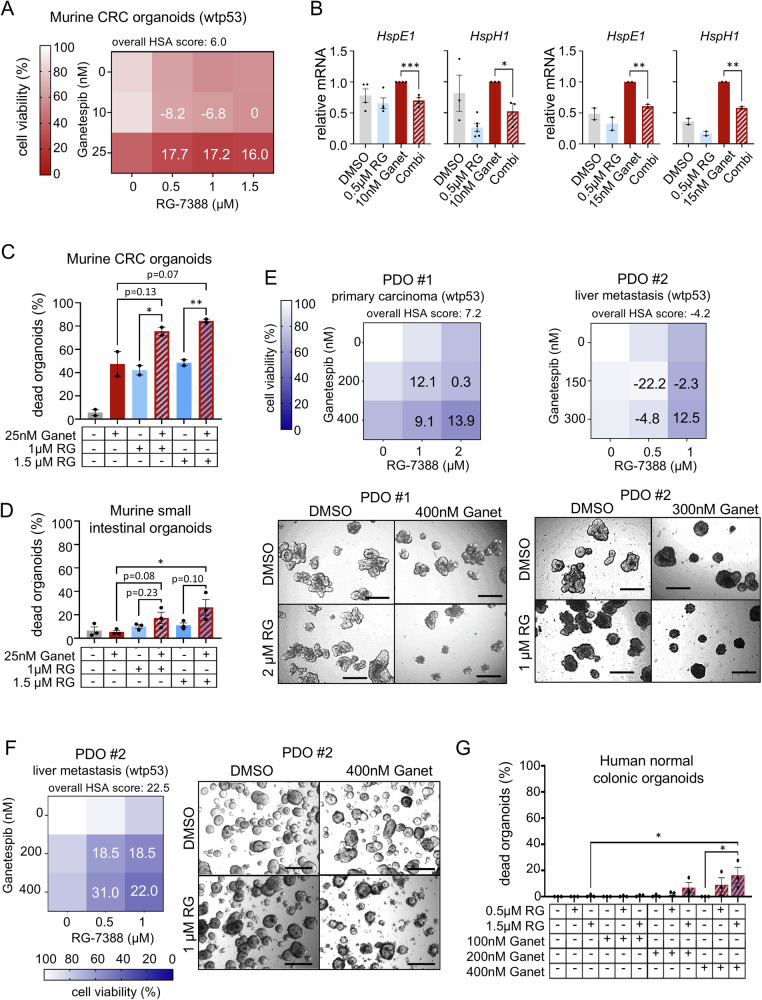
Activated p53 represses the HSF1-mediated heat-shock response in HSP90-based therapies in murine and human colorectal tumor-derived organoids. **A** Cell viability matrix of murine tumor organoids treated with Ganetespib plus RG-7388 combination for 48 h. Organoids were generated from AOM/DSS-treated C57BL6/J mice. Four independent biological replicates with 3 in-plate technical replicates each. **B** mRNA expression levels of HSF1 target genes in murine tumor organoids generated from AOM/DSS mice treated with 10 nM (left) and 15 nM (right) Ganetespib in combination with 0.5 µM RG-7388 for 24 h. qRT-PCRs for the indicated mRNAs normalized to *RPLP0* mRNA. Mean ± SEM from min. 2 independent biological replicates. **C** Quantification of dead tumor-derived organoids treated with indicated drug combination for 48 h. Organoids were generated from AOM/DSS-treated C57BL6/J mice. Mean ± SEM from 2 independent biological replicates. **D** Quantification of normal murine small intestinal mucosa-derived organoids treated with the indicated combinations for 48 h. Mean ± SEM from 3 independent biological replicates. **E** *Top*, Cell viability matrices of p53-proficient (wild-type p53, wtp53) patient-derived organoids (PDOs) treated with Ganetespib, RG-7388, or in combination for 48 or 72 h. A PDO case represents one PDO culture (PDO #). For each PDO culture, four replicates (different passages) with 3 in-plate technical replicates each were measured. Organoids were cultivated and treated as large organoids. *Bottom*, Representative brightfield images of large CRC tumor organoids. Scale bars, 200 μm. **F** Cell viability matrix of PDO #2 culture, cultivated and treated as small organoids with Ganetespib, RG-7388, or in combination for 3 or 5 days. Three replicates (different passages) with 3 in-plate technical replicates each were measured. *Bottom*, Representative brightfield images of small PDOs #2 culture. Scale bars, 200 μm. **G** Quantification of dead organoids via their morphology. Normal human colon mucosa-derived organoids were treated with the indicated combinations for 48 h. Mean ± SEM from 3 independent biological replicates. **A**, **E**, **F** Color scheme represents changes in cell viability. Numbers in the matrix are HSA synergy scores. Synergy scores: <−10 antagonistic; −10 to 10 additive; >10 synergistic. Ganet: Ganetespib, RG: RG-7388. **B**, **C**, **D**, **G** Ganet: Ganetespib, RG: RG-7388. Student’s *t*-test (**B**) or one-way ANOVA (**C**, **D**, **G**), *p ≤ 0.05, **p ≤ 0.01, ***p ≤ 0.001; when ns not significant or *p*-value indicated.

To further strengthen the evidence for p53-mediated improvement of HSP90-based therapies, we generated colorectal tumor-derived organoids from the AOM/DSS mouse model [53]. To this end, we co-treated wild-type p53 organoids with RG-7388 plus Ganetespib and indeed observed decreased cell viability with additive to synergistic effects versus each drug alone (Figs. 3A and S3A). This was accompanied by suppression of HSF1 target genes upon different concentrations of Ganetespib (Fig. 3B). Furthermore, combined drug concentrations impressively increased cell death to up to 80% in murine tumor-derived organoids (Figs. 3C and S3B). Importantly and in contrast, in normal mucosa-derived organoids (generated from murine small intestine) cell death was minimal and did not significantly change (Fig. 3D). We also generated patient-derived organoids (PDOs) from 2 different wtp53 CRC patients, one from a primary tumor (PDO #1) and one from a liver metastasis (PDO #2). Both PDO cultures confirmed the enhanced suppression of cell viability with drug combinations compared to the corresponding single drugs (Fig. 3E, F). Since drug responses can be dependent on the organoid volume [54], we also tested different organoid sizes. Larger, multi-volume organoids (Fig. 3E) as well as single cell-dissociated smaller organoids (Fig. 3F) generated from the same PDO culture (PDO #2) both favorably responded to dual HSF1-HSP90 inhibition with stronger reduced viability and reduction of organoid sizes and numbers compared to single treatments. Notably, normal colonic organoids from patients survived much better using the same combined drug combinations as for PDOs (Figs. 3G and S3C), underlining the tumor selectivity and therapeutic value of dual HSF1-HSP90 pathway inhibition.

### Dual HSF1-HSP90 pathway inhibition reduces colonic tumor progression in a mouse model by remodeling the immune cell composition

Induction of the unwanted heat-shock response is a major problem associated with HSP90 inhibitors in pre-clinical and clinical trials [18, 55–58]. Due to the negative regulatory feedback loop between stress-induced HSP90 that binds back and inhibits HSF1 to normally limit the HSR, inhibition of HSP90 alone inadvertently upregulates HSF1 and the gamut of HSPs like HSP90, HSP40, and multiple HSP70 members, thereby stabilizing client proteins and counteracting the therapeutic effect of HSP90 inhibition. Thus, we examined in the murine AOM/DSS-induced autochthonous colonic tumor model whether the combination treatment would improve HSP90-based therapy in vivo. This model mirrors human colonic tumors at the pathological and molecular level by exclusively generating tumors in the colorectal part of the intestine [59].

**Fig. 4:**
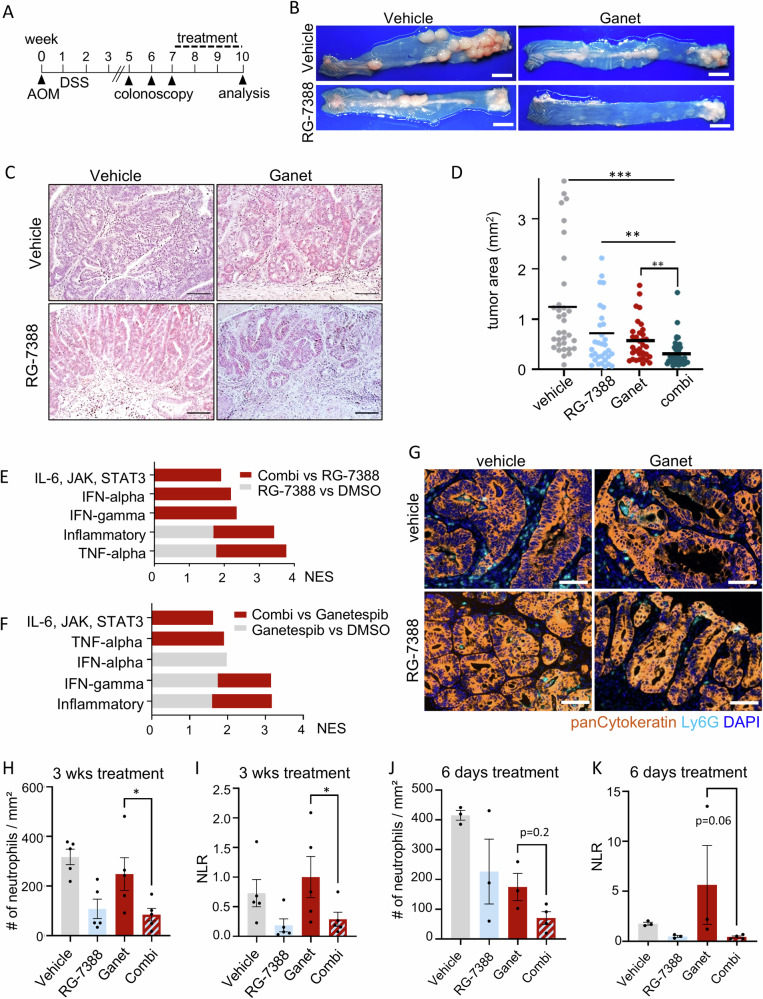
Dual HSF1-HSP90 pathway inhibition reduces colonic tumor progression in a mouse model by remodeling the immune system. **A** Treatment scheme for the p53-proficient AOM/DSS mouse model. After tumor visualization by repeat colonoscopy and tumor scoring for at least one S2-sized tumor and three S1-sized tumors per mouse, mice were treated with single drugs or combination treatment for 19 days. 4 h after the final dose, mice were dissected and analyzed. **B**, **C** Representative images of entire resected colons (**B**) and H&E-stained colon sections (**C**) from single or combination-treated C57BL6/J mice at endpoint as described in (**A**). Mice received 50 mg/kg RG-7388 orally 5× per week, or 50 mg/kg Ganetespib intravenously 2× per week, or both. **B** scale bars, 5 mm. **C** ×20 magnification, scale bars, 1 cm. **D** Tumor surface area from AOM/DSS mice that had received single or combination treatment for 19 days as in (**A**). Cross-sections of Swiss roles were H&E stained (Supplementary Fig. 4A). Tumor areas of all tumors per H&E-stained Swiss role were measured using ImageJ. Bar, mean. vehicle *n* = 35 tumors from 5 mice; RG-7388 and Ganetespib *n* = 33 tumors from 6 mice each; combination *n* = 45 tumors from 9 mice. **E**, **F** GSEA hallmark analysis of RNAseq data from HCT116 cells treated with drug combination or single drugs for 24 h. Enriched pathways are plotted according to NES. Gray bars: Enrichment in (**F**) RG-7388-only or (**G**) Ganetespib-only versus DMSO controls. Red bars: further enrichment with combination treatment relative to single treatment. NES: normalized enrichment score. **G**–**I** Multiplex immunohistochemistry of p53-proficient tumors from AOM/DSS mice receiving drug treatments for 19 days as in (**A**). **G** Representative images of indicated groups. Ly6G staining represents neutrophils, and panCytokeratin represents tumor-epithelial cells. DAPI counter staining. Scale bars, 50 µm. **H** Quantification of Ly6G + CD11b + F4/80− neutrophils using the “Vectra Polaris” platform and the inForm Advanced Image Analysis software. *n* = 5 mice per group. All tumors from stained Swiss roles were analyzed for the indicated treatment groups. **I** Plot of the neutrophil-lymphocyte-ratio (NLR) as a marker of an inflammatory response. A low NLR indicates an inflammatory response. **J**, **K** Multiplex immunohistochemistry of tumor-bearing AOM/DSS mice who received a short drug treatment of 6 days. Quantification of Ly6G + CD11b + F4/80− neutrophils of the indicated treatment groups as in (**H**). DMSO, Ganetespib, and RG-7388 mice *n* = 3 each, combination-treated mice *n* = 4. **C**, **H**–**K** Mean ± SEM. Student’s *t*-test, *p ≤ 0.05, **p ≤ 0.01, ***p ≤ 0.001; ns not significant. Ganet: Ganetespib, RG: RG-7388.

Mice with a defined tumor burden, assessed by colonoscopy, were treated with either RG-7388 plus Ganetespib in combination or each drug alone for 3 weeks (Fig. 4A). Indeed, p53 activation concomitant with HSP90 inhibition strongly reduced the sizes and numbers of established colonic tumors (Fig. 4B). Gross pathological and histological analysis confirmed that combo-treated mice compared to single-treatment mice showed the highest degree of tumor shrinkage and largest reduction in tumor numbers (consistent with tumor prevention) (Figs. 4B–D and S4A). Combination treatment decreased the tumor area by approx. 50%–60% compared to monotherapies (Fig. 4D). Weight loss is a sensitive indicator of the toxicity of cancer therapies. However, the combo-treated mice remained physically active and did not lose weight, indicating a favorable and tolerable toxicity profile in mice (Fig. S4B).

To mechanistically dissect tumor growth reduction upon dual HSF1-HSP90 inhibition, we further analyzed our RNAseq data (Fig. 2E, F). Of note, we observed massive upregulation of inflammatory hallmarks, specifically with combinatorial treatment in human HCT116 cells (Fig. 4E, F). Inflammatory pathways, including IL-6-JAK-STAT3, TNFα, and Interferon signaling, were either exclusively upregulated in response to the combination treatment (red bars) or were further increased in combination treatment compared to single RG-7388 (Fig. 4E) or Ganetespib treatment (Fig. 4F) (gray - red stacked bars). Thus, the GSEA data points to a modulation of immune cell regulatory pathways. This is further supported by the stromal reaction after 3 weeks of combo-treatment, which points to a reactivation of the immune system (Fig. 4C). Of note, Ki67 and CD31 staining failed to show significant changes in proliferation and vessel density (data not shown). Moreover, it is known that cancer-specific alteration of the p53 status affects immune activation and intratumoral composition of immune cell populations [60, 61]. Thus, we performed a multiplex immunohistological (mIHC) analysis to investigate major lymphocytic and myeloid immune cell populations by staining with antibodies for CD4, CD8, Ly6G, CD11b, and F4/80 together with the tumor-epithelial cell marker panCytokeratin (Fig. 4G). The panCytokeratin staining in mIHC analysis (Fig. 4G) reflects the reduction of tumor mass after three weeks of treatment (Fig. 4C). CD4+ T cells, CD8+ T cells, and F4/80+ macrophages displayed no significant changes between treatment groups (data not shown). In contrast, Ly6G + CD11b + F4/80− neutrophils were effectively decreased in combination treatment compared to HSP90-based inhibition alone (Fig. 4G, H). This neutrophil population contains both tumor-associated neutrophils (TAN) and the myeloid-derived suppressor cell subset of neutrophils (PMN-MDSC). The pro-tumorigenic and immunosuppressive role of neutrophils has been increasingly recognized [62]. Reflecting a predominantly myeloid and immunosuppressive character of the tumor microenvironment, a high ratio of neutrophils is an important predictor for a poor clinical outcome in many solid tumors, including CRC [63–68]. Consistent with the observed tumor reduction by our tumor-suppressive combination treatment, we assume that the combination treatment effectively reduced the immunosuppressive intratumoral neutrophil population, suggesting improved intratumoral immunoactivation. A decreased neutrophil-to-lymphocyte-ratio (NLR; Ly6G + CD11b + F4/80− to CD8+) in combo-treated mice compared to Ganetespib treated mice therefore reflects the conversion of the tumor microenvironment into a more immunoactivated state (Fig. 4I). Furthermore, short drug treatments in the AOM/DSS mouse model also tended to decrease neutrophils (Fig. 4J) and the NLR ratio (Fig. 4K), further strengthening that p53 activation in HSP90-based therapies effectively remodels the intratumoral immune cell composition and promotes immune activation.

In sum, we demonstrate a higher pre-clinical efficacy with dual HSF-HSP90 pathway inhibition in an autochthonous mouse colonic tumor model in vivo and confirm that in this synergistic context, HSP90 inhibitors are promising cancer drugs with strong tumor selectivity.

### CDK4/6 inhibition in combination with HSP90 inhibitors impairs viability of p53-deficient cancer cells

The second most common alterations in CRC after APC mutations are bi-allelic *TP53* mutations (typically with LOH of the second allele), affecting over 60% of CRC patients [20] [https://www.cbioportal.org/]. The event of *TP53* mutations typically enables the critical colonic adenoma-to-carcinoma transition into frank invasive cancer and drives tumor aggressiveness [69–71]. Consequently, patients with p53-deficient CRC do not benefit from p53 activators in the context of HSP90 inhibition (see Fig. S1D, HCT116 p53−/− cells). Since we previously established that p53 suppresses HSF1 via a repressive CDKN1A/p21-CDK4/6 axis [36], we hypothesized that direct CDK4/6 inhibitors phenocopy the wtp53 – CDKN1A/p21 activation axis.

**Fig. 5:**
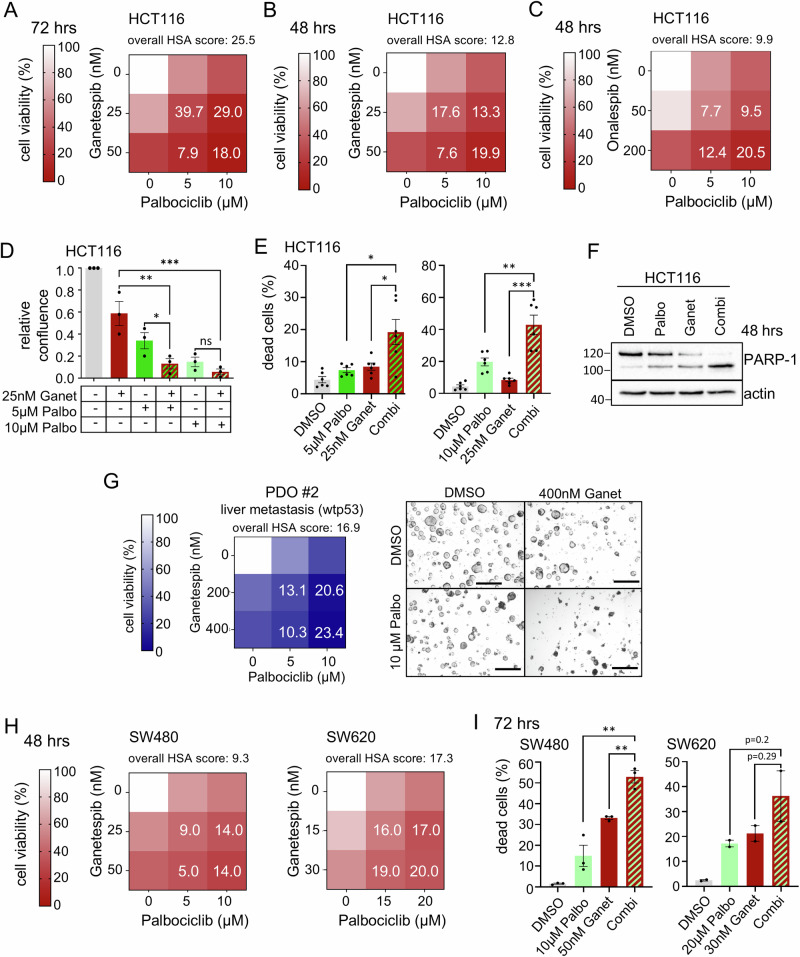
CDK4/6 inhibition in combination with HSP90 inhibitors impairs CRC cell viability independent of the p53 status. **A**–**C** Cell viability matrices of p53-proficient HCT116 cells treated with Palbociclib combined with Ganetespib for **A** 72 h or **B** 48 h; or **C** the HSP90 inhibitor Onalespib combined with Ganetespib for 48 h. *n* ≥ 3 biological replicates. **D** Relative confluence of HCT116 cells after 72 h with the indicated treatments. Cell confluence was analyzed by Celigo imaging cytometry. Confluence relative to DMSO control. Palbo: Palbocliclib, Ganet: Ganetespib. **E** Determination of dead cells. PI/Hoechst/Annexin V staining of HCT116 cells treated for 72 h with Ganetespib and two different Palbociclib concentrations alone or in combination. Percent dead cells (PI+ only, annexin V+ only, and PI+ Annexin V+ cells) were analyzed by Celigo imaging cytometry. **D**, **E** Mean ± SEM from ≥3 biological replicates. Student’s *t*-test, *p ≤ 0.05, **p ≤ 0.01, ***p ≤ 0.001; ns not significant. **F** PARP-1 cleavage in HCT116 cells treated with 10 µM Palbociclib, 50 nM Ganetespib alone or in combination for 48 h. Representative immunoblot from 2 biological replicates. **G** Cell viability matrix of PDO #2 culture, treated as small organoids with the indicated concentrations of Ganetespib, Palbociclib alone or in combination for 72 h. Three replicates (different passages) with 2 in-plate technical replicates each. *Right*, Representative brightfield images after the indicated treatments. Scale bars, 200 μm. **H** Cell viability matrices of p53-deficient SW480 (left) and SW620 (right) human CRC cells treated with Ganetespib - Palbociclib alone or in combination for 48 h. *n* = 3 biological replicates each. **I** Determination of dead cells as in (**E**) for p53-deficient SW480 and SW620 cells treated with Ganetespib and Palbociclib alone or in combination for 72 h. Dead cells were examined by Annexin and PI staining. Mean ± SEM from ≥2 biological replicates. Student’s *t*-test, *p ≤ 0.05, **p ≤ 0.01, ***p ≤ 0.001; ns not significant. **A**–**C**, **G**, and **H** Color scheme represents changes in cell viability. Numbers in the matrix are HSA synergy scores. Synergy scores: <−10 antagonistic; −10 to 10 additive, >10 synergistic.

To this end, we first tested whether CDK4/6 inhibition, in general, contributes to dual HSF1-HSP90 pathway inhibition, focusing on p53-proficient cells. Indeed, in HCT116 cells, Palbociclib, a clinically used CDK4/6 inhibitor, caused a marked reduction of cell viability in combination with Ganetespib (Fig. 5A, B) or Onalespib (Fig. 5C) in HSP90-based therapies. Cell confluence upon Palbociclib plus Ganetespib was strongly decreased, compared to Ganetespib alone (Figs. 5D and S5A). Furthermore, CDK4/6-based dual pathway repression markedly increased cell death, as measured by PI/Annexin V staining (Figs. 5E and S5B) and PARP cleavage by immunoblot (Fig. 5F), compared to single Ganetespib or Palbociclib treatment. Interestingly, in p53-proficient RKO cells, additional CDK4/6 inhibition upon HSP90 inhibition showed only modest differences in cell viability (Fig. S5C–E), cell confluence (Fig. S5F, G), and cell death (Fig. S5H–J). Importantly, however, in patient-derived organoids (PDOs), a clinically relevant translational model system generated from a CRC liver metastasis with wild-type p53, Palbociclib in combination with Ganetespib strongly diminished organoid viability and size (Fig. 5G).

Since over 60% of all CRC patients harbor *TP53* mutations, we reasoned that p53-deficient CRC cells can still efficiently benefit from dual HSF1-HSP90 inhibition by direct CDK4/6 inhibition. To test this, we used SW480 and SW620 cells harboring missense p53 mutations (mutp53). SW620 cells, originally derived from a metastasis, and SW480 cells, derived from the primary adenocarcinoma of the same patient, serve as a model for the advanced CRC tumor stage. Again, we first tested effective therapeutic concentrations for single drugs in cell viability assays (Fig. S5K, L). We chose ICs of 80–70% and approx. 50% for combination treatments. Importantly, in combination, Palbociclib with Ganetespib additively to synergistically reduced cell viability (Figs. 5H and S5M; weaker with Onalespib in Fig. S5N) and increased cell death (Fig. 5I) in all tested concentrations and time points in both p53-deficient cell lines.

**Fig. 6:**
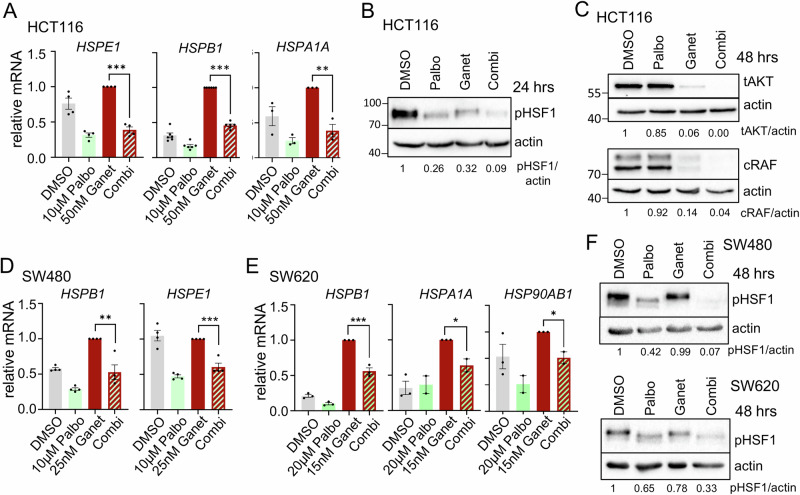
CDK4/6 inhibition in combination with HSP90 inhibitors impairs the counterproductive HSR in p53-deficient human cancer cells. **A** mRNA expression levels of representative HSF1 target genes in HCT116 cells treated as indicated for 24 h. qRT-PCRs, expression levels were normalized to *RPLP0* mRNA. **B**, **C** Immunoblots of HCT116 treated for 24 h (**B**) or for 48 h (**C**) with 50 nM Ganetespib and 10 μM Palbociclib alone or in combination. Representative of 2 biological replicates. Ganet: Ganetespib, Palbo: Palbociclib. **D**, **E** HSF1 target gene expression of mutp53-containing SW480 (**D**) and SW620 (**E**) cells treated with Ganetespib and Palbociclib alone or in combination for 24 h. qRT-PCRs, expression levels were normalized to *RPLP0* or *HPRT1* mRNA. **F** Immunoblots of p53-deficient SW480 and SW620 treated for 48 h with Ganetespib and Palbociclib alone or in combination. SW480 treated with 25 nM Ganet and/or 10 μM Palbo. SW620 were treated with 15 nM Ganet and/or 2.5 μM Palbo. **A**, **D**, **E** Mean ± SEM from ≥3 biological replicates each. Statistics relative to Ganetespib treatment. Student’s *t*-test, *p ≤ 0.05, **p ≤ 0.01, ***p ≤ 0.001; ns not significant. Ganet: Ganetespib, Palbo: Palbociclib.

Mechanistically, inhibition of CDK4/6 by Palbociclib under HSP90 inhibition again repressed HSF1 target genes (Figs. 6A and S6A, B) and lowered the levels of activated pSer326-HSF1 (Fig. 6B) in p53-proficient human CRC cells. Classical HSP90 clients were completely degraded after CDK4/6 inhibition plus Ganetespib (Fig. 6C), again demonstrating a marked HSF1-HSR suppression. Importantly, the same is true for p53-deficient human CRC cell lines (Fig. 6D–F). Palbociclib reduces HSF1 target gene expression (Fig. 6D, E) and pSer326-HSF1 levels (Fig. 6F) in combination treatments compared to HSP90 inhibition alone.

**Fig. 7:**
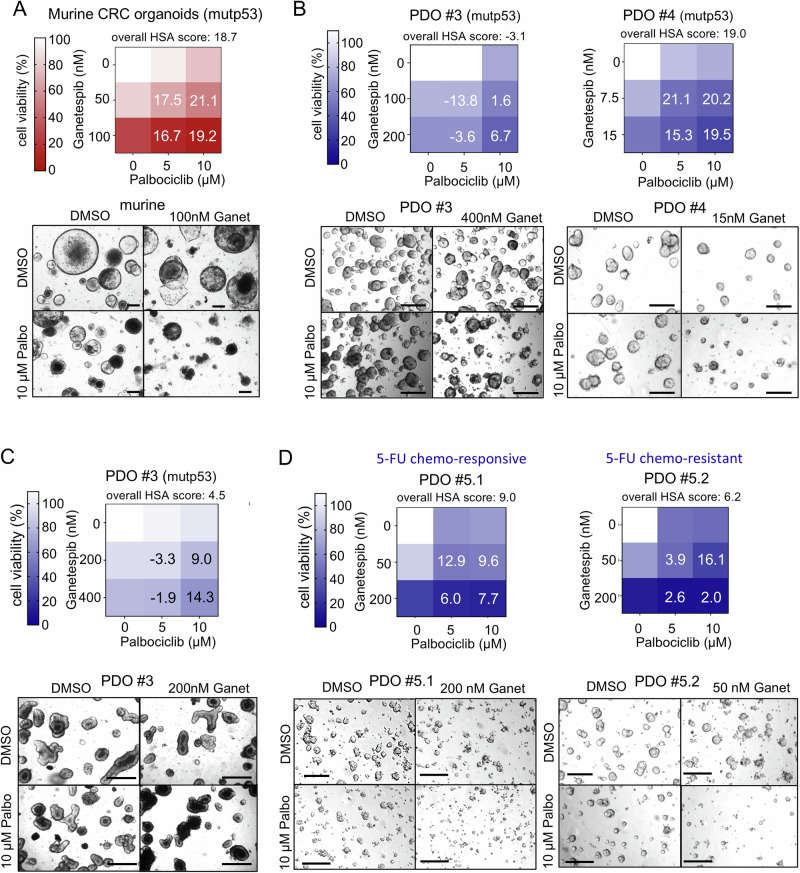
p53-mutant murine organoids and human PDOs synergistically reduce their viability after combined CDK4/6 and HSP90 inhibition. **A** Cell viability matrix of p53-mutant (mutp53) murine CRC tumor organoids treated with Ganetespib and Palbociclib alone or in combination for 72 h. Organoids were generated from AOM/DSS-treated *TP53*^R248Q^ mutant mice [39]. *n* = 3 biological replicates (different passages) with 3 in-plate technical replicates each were measured. **B** Cell viability matrices of *TP53* mutant (mutp53) patient-derived organoids (PDOs) from 2 different patients (PDO #3 and PDO #4) treated with Ganetespib, Palbociclib alone or in combination for 72 h (left) and 96 h (right). PDOs were cultivated and treated as small organoids. **C** Cell viability matrix of mutp53-containing PDO #3 treated with Ganetespib, Palbociclib alone or in combination for 48 h or 72 h. PDOs were cultivated and treated as large organoids. *n* = 5 biological replicates (different passages) with 3 in-plate technical replicates each were measured. **D** Cell viability matrices of a mutp53-haboring matched PDO pair (chemo-responsive and chemo-resistant) from one patient treated with Ganetespib and Palbociclib alone or in combination for 96 h. Both PDOs were cultivated and treated as small organoids. **A**–**D** Color scheme represents changes in cell viability. Numbers in the matrix are HSA synergy scores. Synergy scores: <−10 antagonistic; −10 to 10 additive; >10 synergistic. For each PDO culture *n* ≥ 2 replicates (different passages) with ≥2 in-plate technical replicates each were measured. Bottom, Representative brightfield images of organoids after the indicated treatments. Scale bars, 200 μm.

Next, we could show strong support for this strategy in mutp53 organoids. Humanized *TP53* R248Q/− mutated murine CRC organoids synergistically responded to a Palbociclib-Ganetespib combination compared to single drug treatments, including a reduction of organoid size and numbers (Fig. 7A). In contrast, in normal murine small intestinal organoids, organoid viability was only moderately decreased upon Palbociclib single treatment (Fig. S7A), confirming the low toxicity of this CDK4/6 inhibitor. Dead small intestinal organoids were increased, but combination treatment with Ganetespib did not further increase Palbociclib-induced organoid death (Fig. S7B), again suggesting that our combination strategy with this reversible CDK4/6 inhibitor is well-tolerated.

Next, we analyzed human mutant p53 CRC PDOs derived from 2 different patients. Again, combined CDK4/6 inhibition by Palbociclib plus HSP90 inhibition reduced organoid viability and diminished organoid sizes compared to single drug treatments in single cell-dissociated organoids (Fig. 7B). Also, larger multi-volume organoids responded to dual HSF1-HSP90 inhibition (Fig. 7C). Moreover, we verified the superiority of dual pathway inhibition in a matched pair of chemo-sensitive and chemo-resistant PDOs derived from a third CRC patient under conventional chemotherapy. Even after the tumor had acquired chemoresistance in this patient, dual CDK4/6 inhibition in combination with Ganetespib impaired organoid viability better than single treatment (Fig. 7D). Importantly, in normal human colonic organoids, cell death upon combined drugs was minor (Fig. S7C), confirming the tumor selectivity of this combination in a human background.

In sum, these results strongly support our hypothesis that concomitant CDK4/6 inhibition effectively prevents the unwanted HSR that contributes to therapeutic failure when treating CRC cells with HSP90 inhibitors. This work provides a strategy forward towards improving HSP90-based therapies in CRC independent of its p53 status.

## Discussion

The HSF1-HSP90 system is highly upregulated and activated specifically in cancer cells but not in normal cells [8, 17, 72]. It represents a powerful pro-tumor anti-proteotoxic defense and pro-survival system, targetable with therapeutic selectivity. We and other groups have shown that HSP90 inhibition markedly impairs tumor growth and progression in pre-clinical mouse models in vivo [3–11, 16, 20, 28, 45–48]. Nevertheless, despite these encouraging findings, HSP90 inhibitors, either as monotherapy or combined with conventional chemotherapy, have so far largely failed in clinical oncology trials [6, 7, 29, 31]. One major reason might be the drug-induced abrogation of the HSP90-HSF1 negative feedback loop upon HSP90 inhibition, inadvertently leading to a deleterious rebound HSR with increased chaperone expression [18, 55–58]. Moreover, as a result of HSP90 inhibition, tumor-driving oncoproteins might be further stabilized by alternative chaperones other than HSP90 [32–35]. Hence, there is an urgent clinical need for new strategies to block the counterproductive compensatory HSF1-mediated HSR activation upon HSP90 inhibition. In the AOM/DSS model, pharmacological inhibition or knock-out of HSF1 strongly suppresses colorectal carcinogenesis [73].

Building on our recent finding that HSF1 activity is effectively suppressed by wtp53 via a p53 - CDKN1A/p21 - CDK4/6 - MAPK axis [36], we tested the hypothesis that p53 activation (in case of wtp53, inducing CDKN1A/p21 upregulation) or direct CDK4/6-based cell cycle inhibition (in case of mutp53) provide new opportunities for improving the efficacy of HSP90-based therapy of CRC. Indeed, this is what we observed. Upon HSP90 inhibition, the rebound HSR response was profoundly impaired by concomitant p53 activation in p53-proficient cancer cells or by direct CDK4/6 inhibition in p53-deficient cancer cells. Importantly, while dual HSF1-HSP90 suppression exhibits marked anti-tumoral efficacy, normal tissues and mice showed no significant toxicities. Of note, low concentrations of HSP90 inhibitors (compared to other such studies) in dual HSF1-HSP90 pathway inhibition were sufficient to strongly impair cell growth in colon-derived tumor organoids and in mice. In contrast, the same low dose as monotherapy was insufficient to adequately repress tumor cell growth.

HSP90 inhibitors are widely tested in combination with chemotherapies as a potential path to avoid chemoresistance, with the focus on HSP90 clients rather than on p53 status and HSR activity [47, 50, 74–80]. In contrast, the concept of whether direct non-genotoxic p53 activation in combination with HSP90 inhibition decreases the deleterious HSR in CRC and suppresses cell survival had not previously been tested [74]. One of our previous studies tested first-generation HSP90 inhibitor 17-AAG plus Nutlin-3a (as p53 activator) and found that this combination destabilizes MDMX, an HSP90 client and MDM2-related p53 antagonist, causing inhibition of oncogenic survival [81]. The identified mechanism primarily caused HSP90 client degradation, leading to hyperactivation of the p53 transcriptional program. However, this study did not evaluate HSF1 suppression. Based on our recent work, here we speculate that HSF1 target genes and HSR were also repressed, which then led to more efficient degradation of HSP90 clients such as MDMX. We found that upon dual HSF1-HSP90 pathway inhibition by p53 activation or CDK4/6 inhibition, classic HSP90 clients were even more robustly degraded than upon HSP90 inhibition alone (Figs. 2E and 6C). In support, a recent study found that concomitant CDK4/6 and HSP90 inhibition robustly destabilizes HIF1α (an HSP90 client) and decreases cell viability [82].

In mutp53-haboring cancers, HSP90 inhibition destabilizes mutp53, an HSP90 client, leading to decreased cancer progression [11, 19–21, 45]. On the other hand, it is shown that wtp53 needs HSP90 for its full functional activation [83–87]. Thus, in wtp53-harboring tumors under HSP90 inhibition, wtp53 might not be fully activated by a p53 activator. Nevertheless, wtp53 is still partially active without HSP90 [83–87]. Second, the described MDMX degradation upon HSP90 inhibitor-based combination treatment [81] might compensate for the lowered HSP90-mediated wtp53 activation. Strikingly, our studies show that wtp53 is activated upon Nutlin-3a/Idasanutlin and even hyperactivated upon combination with Ganetespib (e.g., Figs. 2E, F, S2B, C) [36]. Thus, an HSP90 inhibition influences both wtp53 activity and mutp53 protein destabilization.

Interestingly, dual HSF1-HSP90 inhibition strongly activated inflammatory pathways in tumor-epithelial cells (Fig. 4E, F). IL6-JAK-STAT3, TNFα, and Interferon signaling pathways were exclusively upregulated in response to drug combination or further increased in combination treatment versus single treatment. After combined drug exposure in mice, we detected a strong decrease of neutrophil in the remaining tumor tissue stroma, whereas other immune cells remained unchanged. Since this decrease in intratumoral neutrophils was associated with a strong reduction in tumor mass in combination treatments, we conclude that this neutrophil population had predominantly immunosuppressive functions. Intratumoral neutrophils have been increasingly associated with pro-tumorigenic functions [88], including angiogenesis [89] and promotion of metastasis [90]. Thus, a high ratio of neutrophils is frequently correlated with poor survival [63, 64, 91]. The observable loss of neutrophils in the course of our combination therapy, as reflected by a reduced neutrophil-lymphocyte-ratio (NLR), points to a successful conversion towards a less myeloid immunophenotype consistent with intratumoral immune activation. Tumors employ several strategies to escape immune control. Based on our data, we speculate that dual HSF1-HSP90 suppression might target immune suppressive pathways by degrading immunosuppressive enzymes and/or receptors/ligands. Notably, no genes regulating immune suppression are known among HSF1 target genes. A broader analysis would be necessary to identify tumor-epithelial-derived players that contribute to the remodeling of the immune system upon dual HSF1-HSP90 inhibition.

CDK4/6 inhibition phenocopies p53 activation in p53-proficient CRC cells. Importantly, CDK4/6 inhibition also serves as an alternative dual HSF1-HSP90 inhibition strategy in p53-deficient cancer cells, providing a strategy for improving HSP90-based tumor therapies independent of the p53 status. This strategy was successful in p53-deficient patient-derived organoids (PDOs), which strongly responded to CDK4/6 inhibition under HSP90 inhibitors. Moreover, a matched PDO pair from a mutant p53 CRC patient, respectively chemo-sensitive and chemo-resistant upon CRC therapy, impressively showed that dual HSF1-HSP90 inhibition impairs CRC survival independent of therapy status. Still, we propose that patients with p53-proficient tumors might be treated with a non-genotoxic p53 activator rather than a CDK4/6 inhibitor for the extra benefit of hyperactivating the powerful tumor-suppressive p53 programs (Fig. 2F). On the other hand, long-term p53 activation might exert selective pressure on the tumor to acquire *TP53* mutations and favor resistance [92–94]. Proper patient monitoring should, therefore, be performed. If necessary, a subsequent switch to CDK4/6 inhibitors might be a second-line treatment to overcome acquired resistance mediated by *TP53* mutations in HSP90-based therapies. Interestingly, a benefit of CDK4/6 inhibition is the protection of normal cells under chemotherapy, a strategy known as cyclotherapy [95–98]. Thus, CDK4/6-associated HSF1-HSP90 pathway inhibition in combination with chemotherapy might be useful and warrants further investigation.

HSF1 repression is dependent on the p53-p21-CDK4/6 axis, which mainly regulates E2F/RB and DREAM/MuvB target genes [99–103]. We initially provided a link between E2F/RB or DREAM/MuvB targets and the MAPK pathways via MAP3K11/MLK3 because MAP3K11 expression is regulated by p53 and a MAP3K11 siRNA-mediated depletion strongly suppresses HSF1 activity [36]. However, it was pointed out that MAP3K11/MLK3 is not a direct E2F/RB or DREAM/MuvB target, although it is an important MEK1/2-HSF1 regulator. Thus, we hypothesize that other E2F/RB and DREAM/MuvB targets are involved in MLK3 regulation. E2F/RB and DREAM/MuvB target genes are strongly downregulated upon RG-7388 and upon combination (Fig. 2E), and approx. 8% of listed RB/E2F or DREAM/MuvB target genes [101, 102] are proposed to regulate or interact with MLK3 and/or members of MAPK pathways (HARMONIZOME 3.0). Thus, diverse alternative links are likely, and identification of such alternative or additional links between p53-p21-CDK4/6-E2F/RB and/or p53-p21-CDK4/6-DREAM/MuvB to MAPK pathways might provide future opportunities for new drug targets in our proposed combination therapy. MAP kinase inhibition itself might also provide a therapeutic option in HSP90-based cancer therapies. MEK1 is a well-known upstream regulator of HSF1 [104–106]. MEK1 inhibition in HSP90-based therapy was tested in some cancer entities, yielding additive to synergistic effects in, for example, non-small cell lung cancer [105] and melanoma [106], although a possible HSR abrogation again was not studied. Surprisingly, this combination has not yet been tested for CRC. As a caveat, MAP kinases often display a large degree of redundancy and cross-signaling. Some, e.g., AKT and p38 kinases, also regulate HSF1 activity [107–109]. Nevertheless, in principle, systematic drug screens could definitely identify whether alternative pathways also suppress HSF1 activity in HSP90-based therapy in CRC.

In conclusion, HSF1 suppression by direct p53 activation or by CDK4/6 inhibition improves HSP90-based therapies in CRC, independent of the p53 status and in therapy-resistant CRC.

## Supplementary information

Supplementary Information

Original Data Files

check list_RSH

## Data Availability

The RNAseq data from the combinatorial treatments are available at accession number E-MTAB-14877. All other data, datasets used and/or analyzed in the current study are available from the corresponding author upon reasonable request.
