# Supplementary material for: Enhancement of colorectal cancer therapy through interruption of the HSF1-HSP90 axis by p53 activation or cell cycle inhibition
Source: Cell Death Differ. 2025 Apr 9;32(9):1734–49. doi: 10.1038/s41418-025-01502-x (PMC12432187; doi:10.1038/s41418-025-01502-x)
Supplement: Supplementary file 1 — Supplementary Information [file 41418_2025_1502_MOESM1_ESM.pdf]

Supplementary Information corresponding to:

Enhancement of colorectal cancer therapy through interruption of the HSF1-HSP90 axis by p53 activation or cell cycle inhibition

Tamara Isermann [1,2,3], Kim Lucia Schneider [1], Florian Wegwitz [4], Tiago De Oliveira [5], Lena-Christin Conradi [5], Valery Volk [6], Friedrich Feuerhake [6], Björn Papke [2,3], Sebastian Stintzing [3,7], Bettina Mundt [8], Florian Kühnel [8], Ute M. Moll [9] and Ramona Schulz-Heddergott [1,*]

1) Department of Molecular Oncology, University Medical Center Göttingen, Göttingen, Germany
2) Charité – Universitätsmedizin Berlin, Institute of Pathology, Laboratory of Molecular Tumor Pathology and Systems Biology, Berlin, Germany
3) German Cancer Consortium (DKTK); Partner Site Berlin, German Cancer Research Center (DKFZ), Heidelberg, Germany
4) Department of Gynecology and Obstetrics, University Medical Center Göttingen, Göttingen, Germany
5) Department of General, Visceral, and Pediatric Surgery, University Medical Center Göttingen, Germany
6) Institute for Pathology, Hannover Medical School, Hannover, Germany
7) Charité – Universitätsmedizin Berlin, Department of Hematology, Oncology, and Cancer Immunology, Berlin, Germany.
8) Department of Gastroenterology, Hepatology, Infectious Diseases and Endocrinology, Hannover Medical School, Hannover, Germany
9) Department of Pathology, Stony Brook University, Stony Brook, NY

* Corresponding author: ramona.schulz-heddergott@med.uni-goettingen.de

List of Supplementary Information:

Supplementary Methods

Method S1: Histological analysis

Method S2: Cell culture and treatment

Method S3: Cell death assay in human cancer cells

Method S4: Confluence in human cancer cells

Method S5: Preparation and cultivation of murine small intestinal and colonic tumor organoids

Method S6: Organoid morphology and organoid viability

Method S7: Immunoblots

Method S8: Quantitative real-time PCR (qRT-PCR)

Supplementary Tables

Table S1: Reagents and Resources

Table S2: Primers for qPCR

Supplementary Figures

Supplementary Figure S1. Related to Figure 1: Supplementary Figure 1. Dual HSP90-HSF1 inhibition via p53 activation synergistically impairs colorectal cancer cell growth.

Supplementary Figure S2. Related to Figure 2: Dual HSP90-HSF1 inhibition via p53 activation abrogates the HSF1- mediated HSR.

Supplementary Figure S3. Related to Figure 3: Evaluation of Ganetespib single drug response in murine tumor organoids.

Supplementary Figure S4. Related to Figure 4: Targeting the HSF1-HSP90 pathway reduces murine colonic tumor progression.

Supplementary Figure S5. Related to Figure 5: CDK4/6 inhibition in combination with HSP90 inhibitors impairs viability of CRC cancer cells independent of the p53 status.

Supplementary Figure S6. Related to Figure 6: CDK4/6 inhibition in combination with HSP90 inhibitors impairs the HSR in p53-proficient cancer cells.

Supplementary Figure S7. Related to Figure 7: The CDK4/6 inhibitor is well-tolerated in normal murine small intestinal organoids.

Supplementary Methods

Method S1: Histological analysis

Murine formalin-fixed paraffin-embedded (FFPE) tissue was used for Hematoxylin and Eosin (H&E) staining by standard protocol. Tumor areas were determined using H&E stained sections and ImageJ software.

Method S2: Cell culture and treatment

RKO, HCT116+/+, HCT116-/- and SW480 cells were cultured in RPMI 1640 supplemented with 10 % fetal bovine serum, glutamine and penicillin/streptomycin. SW620 cells were cultured in Leibovitz medium supplemented with 10 % fetal bovine serum and penicillin/streptomycin. All cells besides SW620 grew in a humidified atmosphere at 37°C with 5 % CO_2. SW620 cells grew under 0 % CO_2. RG-7388 (Merck), Palbociclib (Sigma) and Ganetespib (Syntha Pharmaceuticals) were dissolved according to manufacturer's guidelines and used as indicated. Cell lines are tested regularly for mycoplasma contamination. Authentication of cell lines were done by the Deutsche Stammsammlung für Mikroorganismen und Zellkulturen (DSMZ).

Method S3: Cell death assay in human cancer cells

Propidium iodide (PI), Hoechst 33342 (Hoechst) and FITC Annexin V staining was performed for quantification of cell death. Cells were seeded in black clear-bottom 96-well plates. Following experimental treatments at end point, 7 µg/mL PI, 70 µg/mL Hoechst and 0.6 µg/ mL Annexin V prediluted in Annexin V binding buffer were added to the medium. Following incubation for 20 min, the Celigo Imaging Cytometer (Nexcelom) was used for the acquisition and analysis of fluorescence images. Definition for staining: 'dead cells' are all cells stained with Annexin V and/or PI; only Annexin V indicates early apoptosis; Annexin V + PI indicates late apoptosis; only PI indicates cell death other than apoptosis.

Method S4: Confluence in human cancer cells

Cells were seeded in 96-well plates (Corning) and treated as described. The confluence of living cells was measured daily using the Celigo Imaging Cytometer and the Nexcelom Software v5.0.0.0.

Method S5: Preparation and cultivation of murine small intestinal and colonic tumor organoids

In brief, 10-week old C57BL/6J mice were subjected to AOM/DSS induction of colonic tumors as described above. Once mice had developed tumors, organoids were generated from normal small intestinal epithelium and from colonic tumors. To this end, the small intestinal tissue was incubated in EDTA/PBS and colonic tumor tissue was incubated in collagenase. Crypts were cultivated in Matrigel and appropriate medium at 37°C with 5 % CO_2. Small intestinal organoid medium: advanced DMEM F-12, supplemented with 10 % Rspondin-1 conditioned medium, 20 % Noggin conditioned medium, 50 ng/mL rmEGF, 80 µM N-Acetyl-L-Cysteine, N2, B-27, HEPES, Penicillin/Streptomycin and GlutaMAX. Colonic organoid medium: advanced DMEM F-12, supplemented with 50 % Wnt3a conditioned medium, 10 % Rspondin-1 conditioned medium, 20 % Noggin conditioned medium, 200 ng/mL rmEGF, 80 µM N-Acetyl-L-Cysteine, 10 mM Nicotinamide, 500 nM A83-01, 5 µM CHIR 99021, 3.4 µg/mL ROCK inhibitor, N2, B-27, HEPES, Penicillin/Streptomycin and GlutaMAX.

Method S6: Organoid morphology and organoid viability

Murine organoids and PDOs were seeded in 96-well plates (Corning) as at least two in-plate replicates and treated as described. Brightfield images were taken by the Celigo Imaging Cytometer (Nexcelom). For quantification of dead organoids, brightfield images of treated organoids were used. Dead tumor colonic organoids and small intestinal organoids were counted by visual inspection. A dead organoid was classified as one with no intact outer membrane and dark, extruded cells in the organoid lumen.

Organoid viability was measured with CellTiter-Glo® 3D assay (Promega) accordingly to the manufacturer's guidelines. Synergy scores were calculated using the synergyfinder.org web application due to organoid viability where some single dose treatments at low concentration reached a viability above 100% relative to the DMSO control. Synergy scores: < -10 is antagonistic, -10 to 10 is additive, and > 10 is synergistic.

Method S7: Immunoblots

RIPA buffer (1 % TritonX-100, 1 % Desoxycholate, 0.1 % SDS, 150 mM NaCl, 10 mM EDTA, 20 mM Tris-HCl pH7.5 and complete protease inhibitor mix, Roche) was used to prepare protein lysates. Lysates were sonicated and centrifuged, followed by BCA protein assay (Pierce) to determine protein concentrations. For SDS-polyacrylamide gel electrophoresis, equal amounts of protein were run and transferred onto nitrocellulose membranes (Millipore). The membranes were blocked with 5 % milk and incubated with the following antibodies: PARP-1, AKT and cRAF (Cell Signaling), phospho-Ser326 HSF1 and beta-Actin (Abcam). More details on antibody dilutions in Table 1. Densitometric measurements for quantification of immunoblot bands were done using the gel analysis software Image Lab™ (BioRad) and normalized to loading controls.

Method S8: Quantitative real-time PCR (qRT-PCR)

RNA from cells or organoids was isolated with Trizol according to the manufacturer's guidelines (Invitrogen/Thermo Fisher Scientific). Equal amounts of RNA were transcribed with reverse-transcription (M-MuLV Reverse Transcriptase, NEB). Quantitative real-time PCR (qRT-PCR) analysis was performed using a SYBR green-based qPCR Master-Mix (75 mM Tris-HCl pH 8.8, 20 mM $(NH_4)_2SO_4$, 0.01 % Tween-20, 3 mM $MgCl_2$, SYBR Green 1 : 80,000, 0.2 mM dNTPs, 20 U/ml Taq-polymerase, 0.25 % TritonX-100, 300 mM Trehalose). CT values of the genes of interest were normalized to *RPLP0* mRNA. Mean ± SEM of 2 or more independent experiments, pipetted at least in duplicates. Primers are specified in Table 2.

Supplementary Tables

Table S1: Reagents and Resources

REAGENT or RESOURCE	SOURCE	IDENTIFIER
**Antibodies**		
Rabbit monoclonal phospho-Ser326-HSF1	Abcam	Cat# ab76076; RRID:AB_1310328
Rabbit polyclonal anti-HSF1 (H-311)	Santa Cruz	Cat# sc-9144; RRID:AB_2120276
Rabbit polyclonal anti-AKT	Cell Signaling	Cat# 9272; RRID:AB_329827
Mouse monoclonal anti-beta-actin	Abcam	Cat# ab6276; RRID:AB_2223210
Rabbit polyclonal anti-c-Raf	Cell Signaling	Cat# 9422; RRID:AB_390808
Rabbit polyclonal anti-PARP-1	Cell Signaling	Cat# 9542; RRID:AB_2160739
**Chemicals, Peptides, and Recombinant Proteins**		
AOM (Azoxymethane)	Sigma Aldrich	Cat# A5486
DSS (Dextran sodium sulfate)	MP Biomedicals	Cat# 160110
Trizol	Invitrogen	Cat# 15596026
PD 0332991 isethionate (Palbociclib)	Sigma Aldrich	Cat# PZ0199
Idasanutlin (RG-7388)	Merck	Cat# ADV947324054-5MG
Idasanutlin (RG-7388) for *in vivo*	MedChem Tronica	Cat# HY-15676
Ganetespib	Provided by Synta Pharmaceuticals	N/A
CellTiter-Glo Luminescent Cell Viability	Promega	Cat# G7571
CellTiter-Glo 3D Cell Viability	Promega	Cat# G9682
Propidium Iodide	Sigma Aldrich	P4864-10ML
Hoechst 33342	Invitrogen	Cat# H3570
FITC Annexin V	Biolegend	Cat# 640945
Annexin V binding buffer	Biolegend	Cat# 422201
**Experimental Models: Cell Lines**		
HCT116	ATCC	Cat# ATCC® CCL-247™
RKO	ATCC	Cat# ATCC ® CRL-2577™
HCT116 p53-/-	Bunz et al., 1998.	B. Vogelstein, Baltimore
HCT116 p53+/+	Bunz et al., 1998.	B. Vogelstein, Baltimore
SW480	DSMZ	Cat# ACC 313
SW620	ATCC	Cat# ATCC ® CRL-227™
**Experimental Models: Organisms/Strains**		
Mouse: C57BL/6J	N/A	Jax strain# 005304
**Software and Algorithms**		
ImageJ software	Open source	https://imagej.net/Welcome
GraphPadPRISM®	Graphpad Software, Inc.	https://www.graphpad.com/
Image Lab™ Software	Biorad	http://www.bio-rad.com/de-de/product/image-lab-software
Celigo Imaging Cytometer	Nexceloim Bioscience	https://www.nexcelom.com/nexcelom-products/cellometer-and-celigo-image-cytometers/celigo-imaging-cytometer/
ZEN	Zeiss	https://www.zeiss.de/mikroskopie/produkte/mikroskopsoftware/zen.html

Table S2: Primers for qPCR.

Gene	Origin	Forward	Reverse
**qPCR**			
HSPA1A	Human	5'-TCAAGGGCAAGATCAGCGAG	5'-TGATGGGGTTACACACCTGC
HSPH1	Human	5'-ACTGCTTGTTCAAGAGGGCTGTGA	5'-AACATCCACACCCACACACATGCT
HSPE1	Human	5'-CGCTGTTGGATCGGGTTCTA	5'-GGTGCCTCCATATTCTGGGA
HSPB1	Human	5'-GGAGTGGTCGCAGTGGTTAG	5'-ATGTAGCCATGCTCGTCCTG
HSP90AB1	Human	5'-GCAAGCCTACGTTGCTCACT	5'-GCGAATCTTGTCCAAGGCATCA
RPLP0 (36B4)	Human	5'-GATTGGCTACCCAACTGTTG	5'-CAGGGGCAGCAGCCACAAA
HspH1	Mouse	5'-AGACCATCGCCAACGAGTTC	5'-ACATGACCTTTATTCCCACGC
HspE1	Mouse	5'-GGAGTGCTGCCGAAACTGTA	5'-CCAACTTTCACACTGACAGGC
Hprt1	Mouse	5'-GCTTCCTCCTCAGACCGCTT	5'-CCAGCAGGTCAGCAAAGAACT
Rplp0 (36B4)	Mouse	5'-GCAGATCGGGTACCCAACTGTT	5'-CAGCAGCCGCAAATGCAGATG

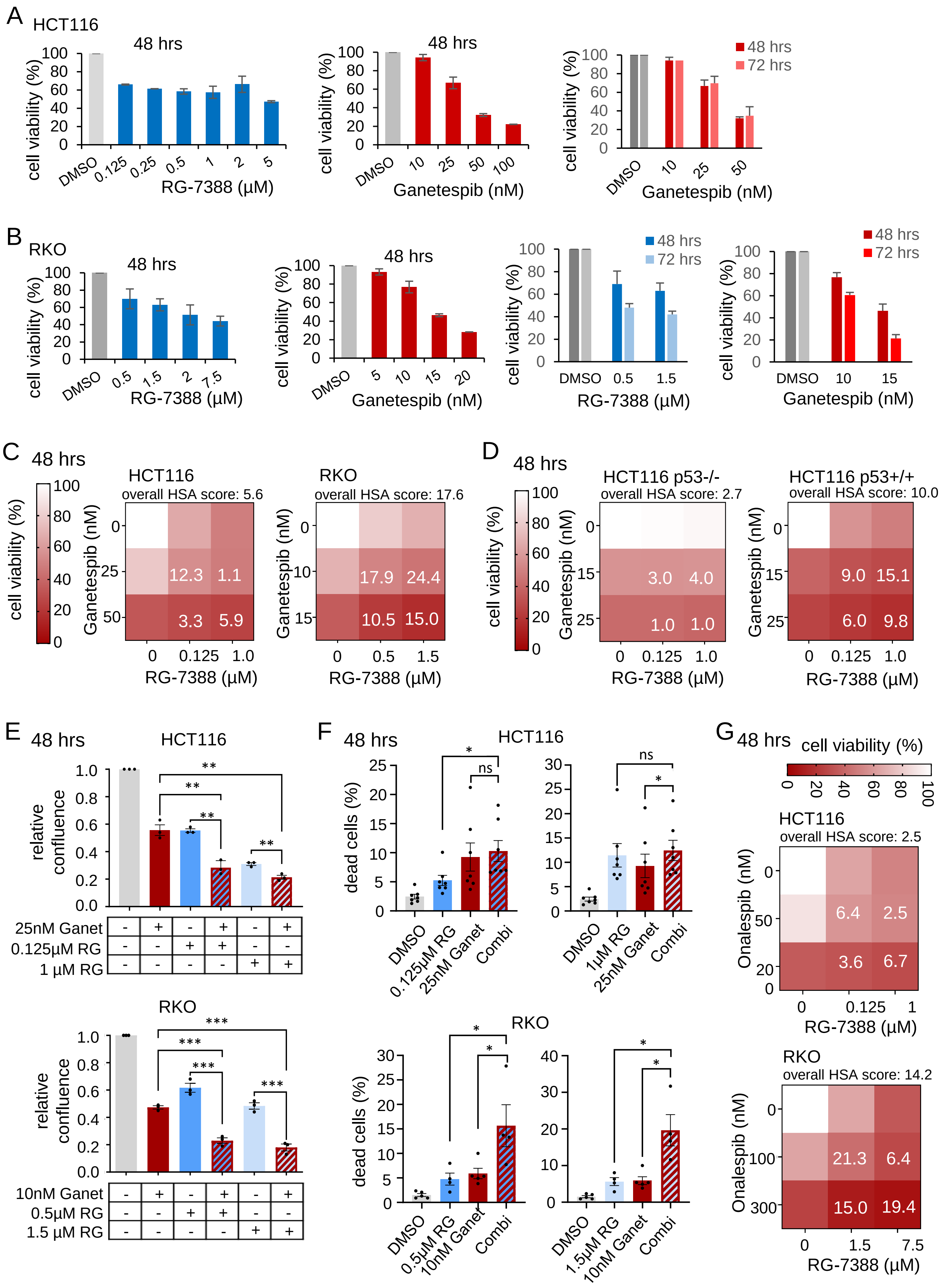

Supp Figure 1

Supplementary Figure 1. Dual HSP90-HSF1 inhibition via p53 activation synergistically impairs colorectal cancer cell growth

(A, B) Cell viability matrices of p53-proficient (A) HCT116 and (B) RKO cells treated with Ganetespib or RG-7388 for single drug reponses at indicated time points and concentrations. Mean ± SEM from ≥ 3 biological replicates.

(C, D) Cell viability matrices of (C) HCT116 and RKO cells and (D) isogenic HCT116 p53-/- and HCT116 p53+/+ cells treated with Ganetespib / RG-7388 combinations for 48 hrs at the indicated concentrations. ≥ 3 biological replicates.

(E) Relative confluence of HCT116 (top) and RKO (bottom) cells treated for 48 hrs. Cell confluence was analyzed by Celigo imaging cytometer. Confluence relative to DMSO control, set at value 1.

(F) Induction of cell death. PI/Hoechst/Annexin V staining of HCT116 (top) and RKO (bottom) cells treated for 48 hrs with Ganet plus increasing concentrations of RG-7388. Percent dead cells include PI+ only, annexin V+ only and PI+ Annexin V+ cells and were analyzed using a Celigo imaging cytometer.

(G) Cell viability matrices of HCT116 and RKO cells treated with Onalespib – RG 7388 combinations for 48 hrs at the indicated concentrations. ≥ 2 biological replicates.

(E-F) Mean ± SEM from ≥ 3 biological replicates, Student's t-test, p*≤ 0.05, p**≤ 0.01, p***≤ 0.001; ns, not significant. Ganet: Ganetespib, RG: RG-7388.

(C, D, G) Color scheme represents changes in cell viability. Numbers within the matrix indicate the HSA synergy score. Synergy scores: < -10 antagonistic; -10 to 10 additive; > 10 synergistic.

A

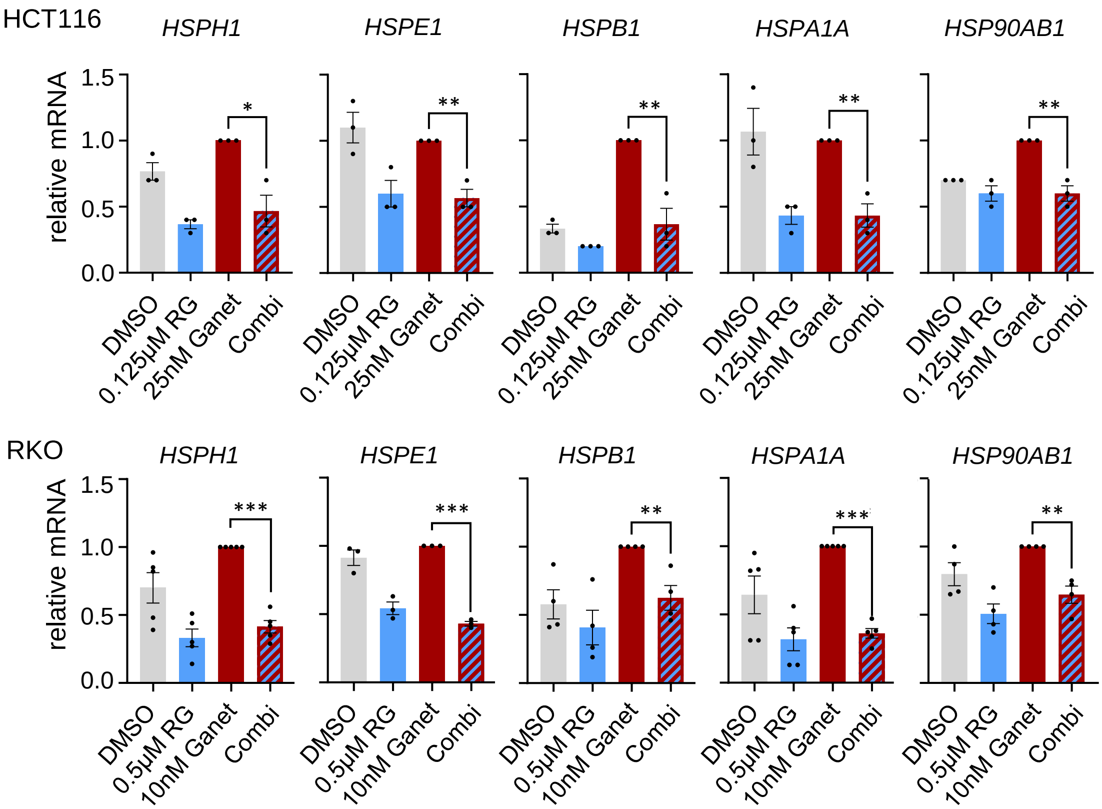

B

HCT116

Gene name	Log2FC RG vs DMSO	pval RG vs DMSO	Log2FC combi vs Ganet	pval combi vs Ganet
CDKN1A	4.46	2.58E-89	3.62	1.41E-59
MDM2	3.75	2.63E-83	3.49	1.38E-73
ZMAT3	2.78	2.31E-47	2.52	9.67E-40
GADD45A	2.02	6.73E-06	2.11	2.60E-06
BTG2	3.49	9.94E-68	2.60	4.95E-39
SESN1	2.64	6.21E-48	2.11	1.26E-32
SESN2	2.00	1.24E-06	1.90	3.76E-06

C

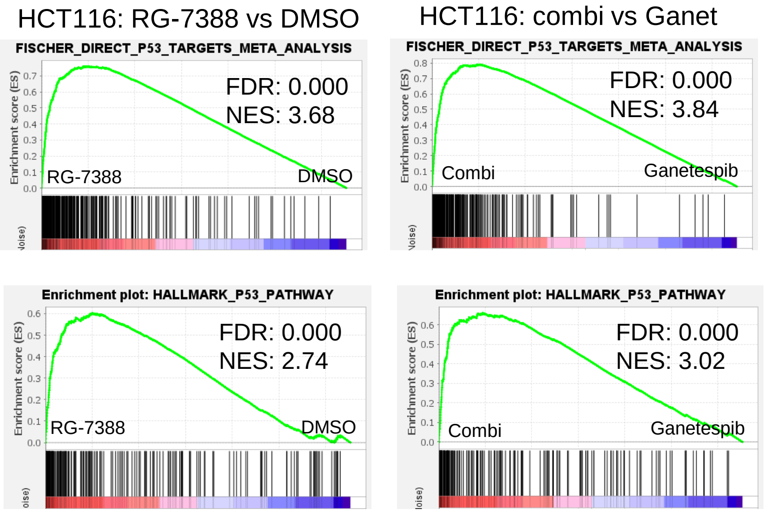

D

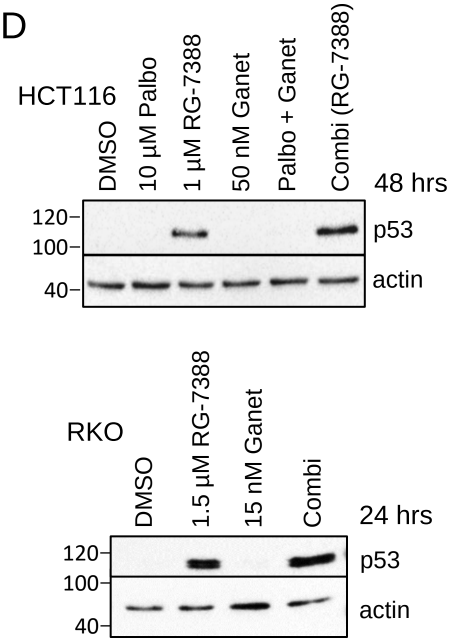

Supp Figure 2

Supplementary Figure 2. Dual HSP90-HSF1 inhibition via p53 activation abrogates the HSF1- mediated HSR

(A) mRNA expression levels of HSF1 target genes in HCT116 (top) and RKO (bottom) cells treated for 24 hrs with low concentrations of Ganetespib plus RG-7388. qRT-PCRs for the indicated mRNAs normalized to RPLP0 mRNA. Mean ± SEM from ≥ 3 biological replicates. Student's t-test, $p^{*} \leq 0.05$, $p^{**} \leq 0.01$, $p^{***} \leq 0.001$; ns, not significant. Ganet: Ganetespib, RG: RG-7388.

(B) Selected p53 target genes from the DEseq list in HCT116 for indicated treatment.

(C) GSEA enrichment plot for two different p53 target gene sets in HCT116 cells treated with RG-7388 alone versus DMSO (left) and with drug combination (1µM RG-7388 plus 50nM Ganetespib) versus Ganetespib alone (50 nM) (right).

(D) Immunoblots for HCT116 and RKO cells treated as indicated. Blots were probed for p53. Actin, loading control. For RKO cells, actin corresponding to p53 is in parallel shown in Figure 2C for pHSF1 staining in RKO cells, because p53 and pHSF1 were processed in parallel on the same membrane, and consequently actin as loading control is shown twice.

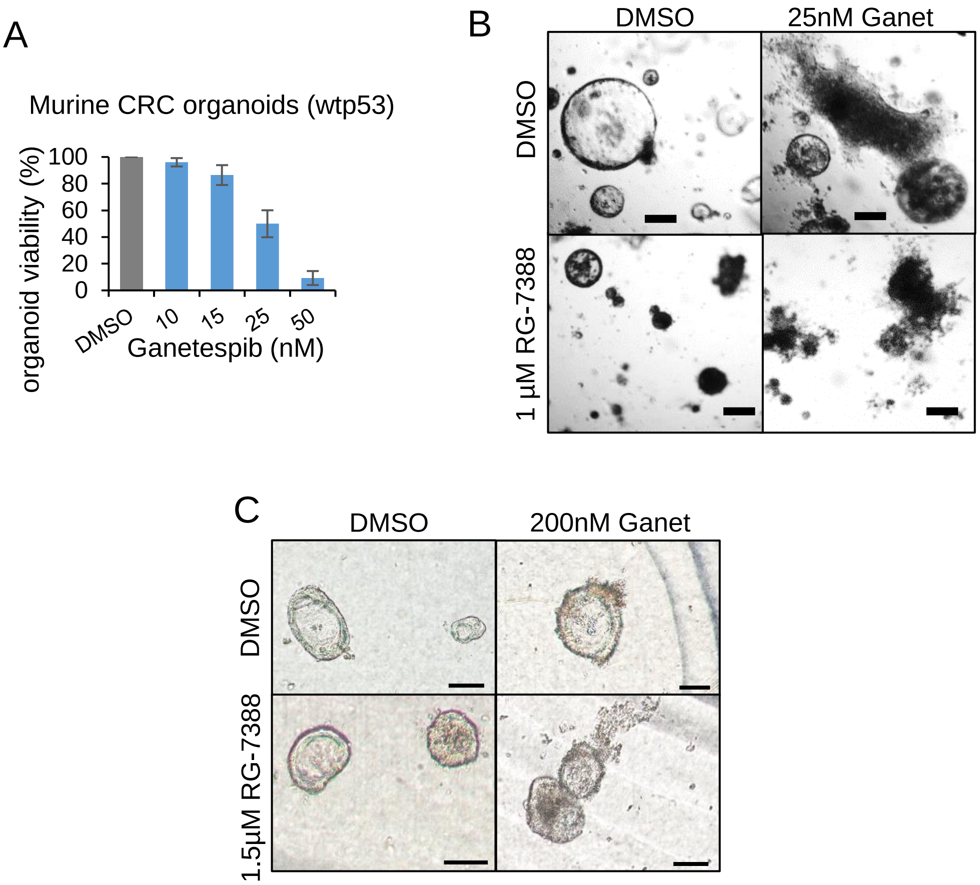

Supp Figure 3

Supplementary Figure 3. Evaluation of Ganetespib single drug response in murine tumor organoids.

(A) Organoid viability assay of murine tumor organoids treated with Ganetespib of the indicated concentrations for 48 hrs. Organoids were generated from AOM/DSS treated C57BL6/J mice. Mean ± SEM from 5 independent biological replicates with 3 in-plate technical replicates each.

(B) Murine tumor-derived organoids treated with indicated drug combination for 48 hrs. Organoids were generated from AOM/DSS treated C57BL6/J mice. Representative brightfield images. Scale bars, 100 µm. Ganet: Ganetespib, RG: RG-7388.

(C) Representative brightfield images of normal human colon mucosa-derived organoids treated with the indicated drug combination for 48 hrs. Scale bar, 100 µm. Ganet: Ganetespib.

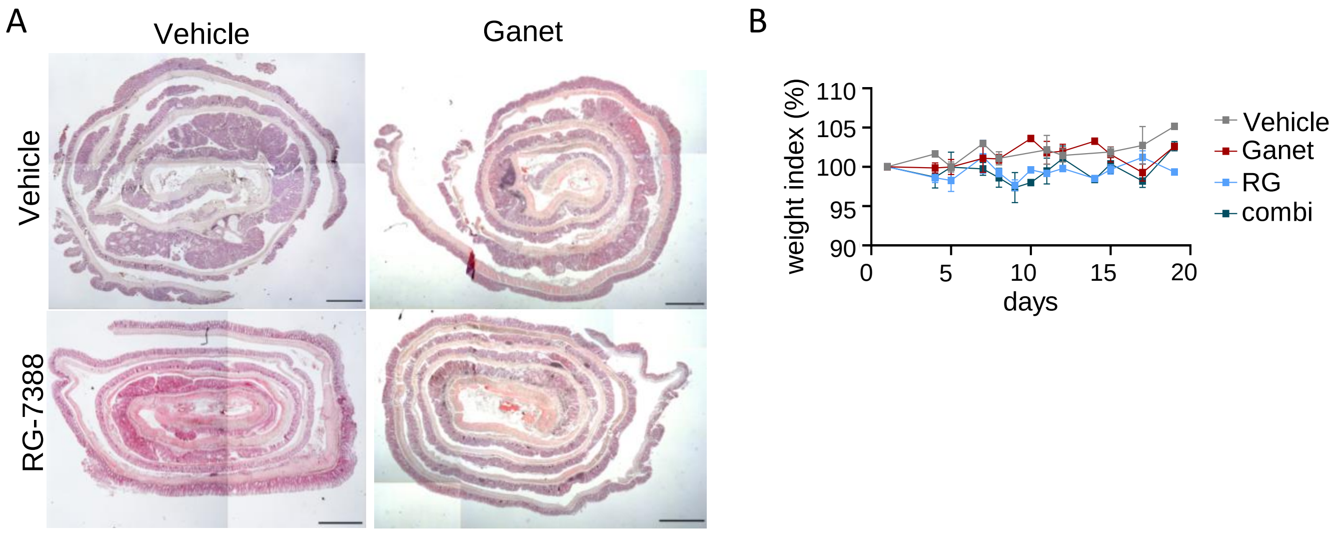

Supp Figure 4

Supplementary Figure 4. Targeting the HSF1-HSP90 pathway reduces murine colonic tumor progression

(A) Representative images of H&E-stained cross-sections of rolled-up resected full-length colons (called swiss role) from single or combination treated C57BL6/J mice at endpoint. Mice received 50 mg/kg RG-7388 orally 5x per week, or 50 mg/kg Ganetespib intravenously 2x per week, or both. x2.5 magnification, scale bar 2 mm.

(B) Lack of weight change in single or combination treated mice over the duration of the drug treatment for 19 days. Mean ± SEM from ≥ 5 mice per group. The weight of each mouse at the start of treatment was set to 100%. Y-axis starts at 90%. Ganet: Ganetespib, RG: RG-7388.

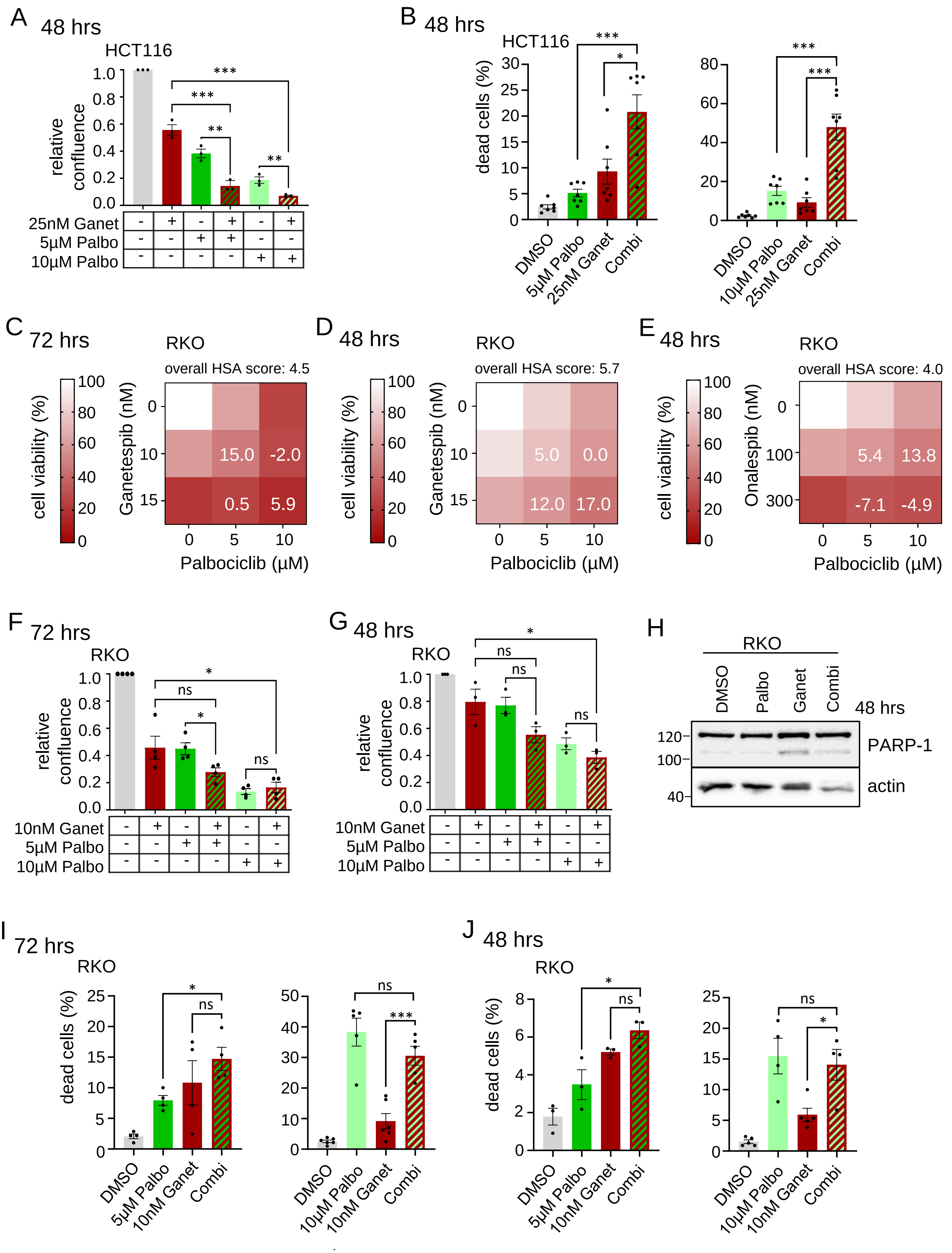

Supp Figure 5

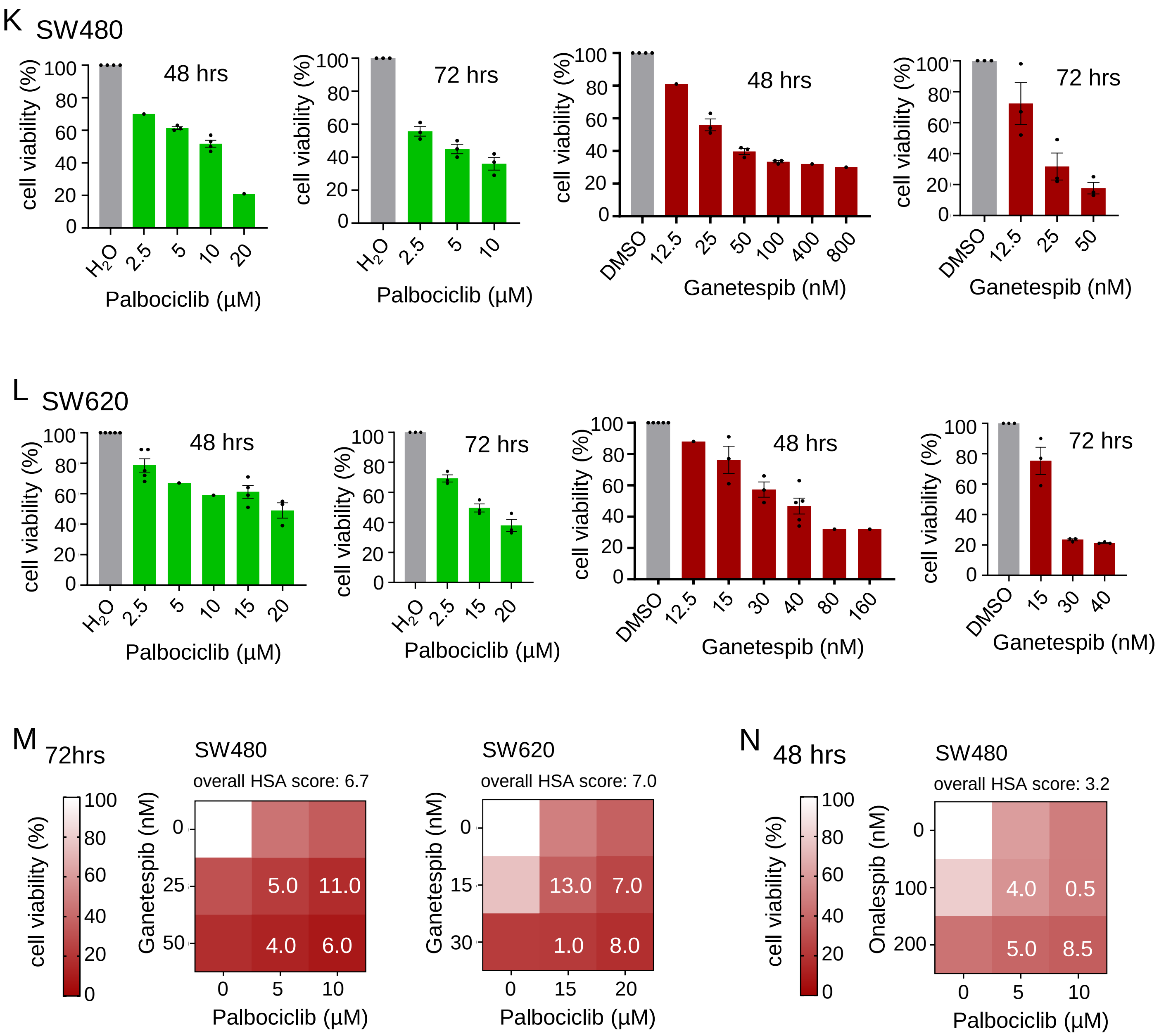

Extended Supp Figure 5

Supplementary Figure 5. CDK4/6 inhibition in combination with HSP90 inhibitors impairs viability of CRC cancer cells independent of the p53 status

(A) Relative confluence of p53-proficient HCT116 cells treated with Ganetespib alone or in combination with Palbociclib for 48 hrs, analyzed by Celigo imaging cytometry. Confluence relative to DMSO control. Palbo: Palbocliclib, Ganet: Ganetespib.

(B) Determination of dead cells treated with Ganetespib and two different Palbociclib concentrations alone or in combination for 48hrs. Percent dead cells (PI+ only, annexin V+ only and PI+ Annexin V+ cells) were analyzed using by Celigo imaging cytometry.

(C-E) Cell viability matrices of RKO cells treated with Ganetespib and Palbociclib alone or in combination for (C) 72 hrs or (D) 48 hrs; or with Onalespib and Palbociclib alone or in combination for 48 hrs (E).

(F, G) Relative confluence of RKO cells treated similar as in (A).

(H) PARP-1 cleavage in RKO cells treated with 10 µM Palbociclib, 15 nM Ganetespib alone or in combination for 48 hrs. Representative immunoblot from 2 biological replicates.

(I, J) Determination of dead cells as in (B). p53-proficient RKO cells were treated for 72 hrs (I) or 48 hrs (J) with Ganetespib and two different Palbociclib concentrations alone or in combination. Dead cells were examined by Annexin and PI staining.

(K, L) Cell viability assays of (K) SW480 and (L) SW620 cells treated with Ganetespib or Palbociclib for single drug responses at indicated time points and for indicated concentrations. Mean ± SEM. Biological replicates are indicated by dots within the bars.

(M) Cell viability matrices of p53 mutant SW480 and SW620 cells treated with Ganetespib – Palbociclib alone or in combination as in (C) for 72 hrs.

(N) Cell viability matrix of SW480 cells treated for 48 hrs with Onalespib and Palbociclib alone or in combination.

(A, B, F, G, I, J) Mean ± SEM from ≥ 3 biological replicates. Student's t-test, $p^* \leq 0.05$, $p^{**} \leq 0.01$, $p^{***} \leq 0.001$; ns, not significant. Ganet: Ganetespib, Palbo: Palbociclib.

(C, D, M) n ≥ 3 biological replicates each, (E, N) n = 2 biological replicates each.

(C-E, M, N) Color scheme represents changes in cell viability. Numbers in the matrix are HSA synergy scores. Synergy scores: < -10 is antagonistic, -10 to 10 is additive, > 10 is synergistic.

A

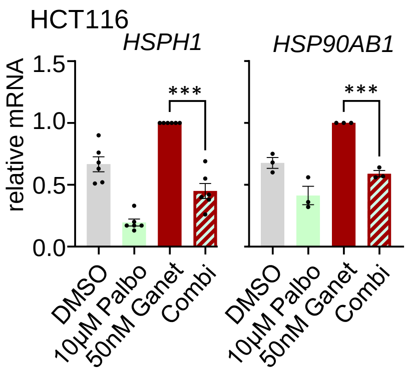

B

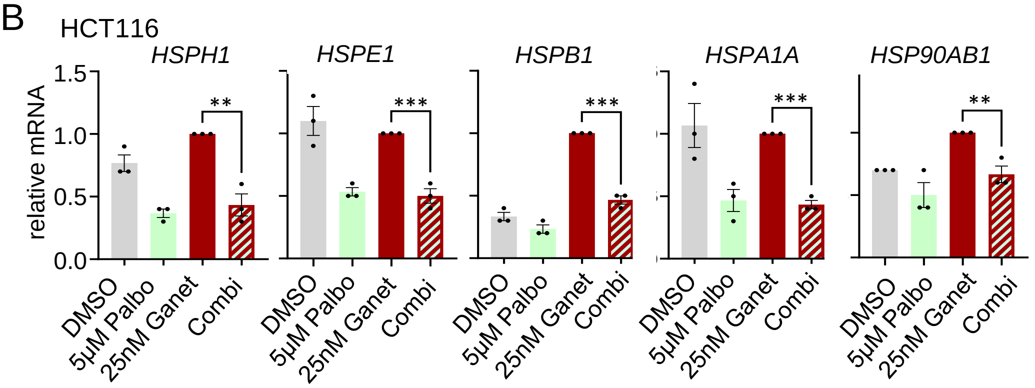

Supp Figure 6

Supplementary Figure 6. CDK4/6 inhibition in combination with HSP90 inhibitors impairs the HSR in p53-proficient cancer cells

(A, B) mRNA expression levels of representative HSF1 target genes in HCT116 cells treated for 24 hrs with the indicated concentrations of Ganetespib and Palbociclib. qRT-PCRs, expression levels normalized to RPLP0 mRNA. Mean ± SEM from ≥ 3 biological replicates each. Student's t-test, p*≤ 0.05, p**≤ 0.01, p***≤ 0.001; ns, not significant. Ganet: Ganetespib, Palbo: Palbociclib.

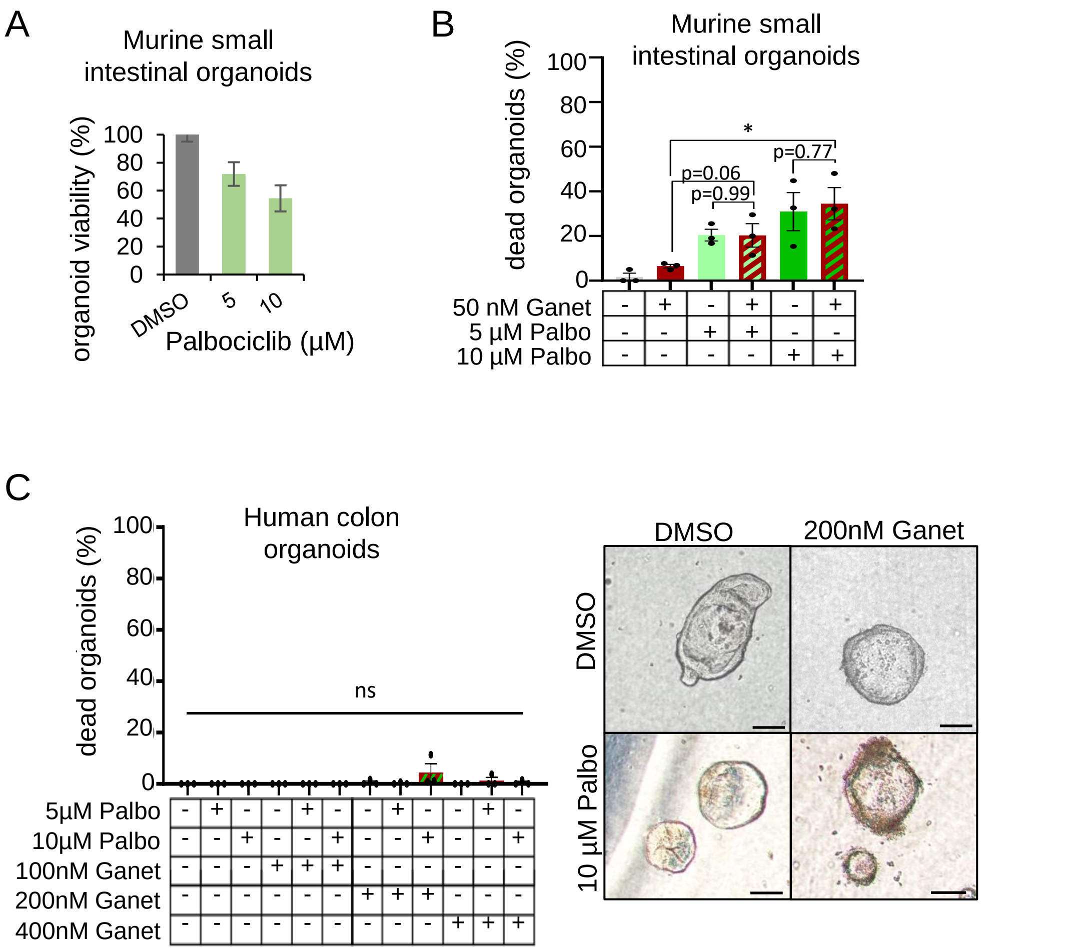

Supp Figure 7

Supplementary Figure 7. The CDK4/6 inhibitor is well-tolerated in normal murine small intestinal organoids.

(A) Organoid viability of murine small intestinal organoids treated with Palbociclib alone at indicated concentrations for 48 hrs. Organoid viability relative to DMSO control. n = 3 biological replicates (different passages) with 3 in-plate technical replicates each were measured. Mean ± SEM.

(B) Determination of dead organoids. PI/Hoechst/Annexin V staining of normal small intestinal organoids treated for 48 hrs with indicated Ganetespib and Palbociclib concentrations alone or in combination. Percent dead organoids (PI+ only, annexin V+ only and PI+ Annexin V+ cells) were analyzed by Celigo imaging cytometry.

(C) Left, Quantification of normal human colon mucosa-derived organoids treated with the indicated combinations for 48 hrs. Mean ± SEM from 3 independent biological replicates. One-way ANOVA, ns: not significant. Right, Representative brightfield images. Scale bar, 100 µm. Ganet: Ganetespib, Palbo: Palbociclib.
