# Supplementary material for: Enhancement of colorectal cancer therapy through interruption of the HSF1-HSP90 axis by p53 activation or cell cycle inhibition
Source: Cell Death Differ. 2025 Apr 9;32(9):1734–49. doi: 10.1038/s41418-025-01502-x (PMC12432187; doi:10.1038/s41418-025-01502-x)

Supplemental Material

Original data files corresponding to:

Enhancement of colorectal cancer therapy through interruption of the HSF1-HSP90 axis by p53 activation or cell cycle inhibition

Tamara Isermann [1,2,3], Kim Lucia Schneider [1], Florian Wegwitz [4], Tiago De Oliveira [5], Lena-Christin Conradi [5], Valery Volk [6], Friedrich Feuerhake [6], Björn Papke [2,3], Sebastian Stintzing [3,7], Bettina Mundt [8], Florian Kühnel [8], Ute M. Moll [9] and Ramona Schulz-Heddergott [1,*]

1) Department of Molecular Oncology, University Medical Center Göttingen, Göttingen, Germany
2) Charité – Universitätsmedizin Berlin, Institute of Pathology, Laboratory of Molecular Tumor Pathology and Systems Biology, Berlin, Germany
3) German Cancer Consortium (DKTK); Partner Site Berlin, German Cancer Research Center (DKFZ), Heidelberg, Germany
4) Department of Gynecology and Obstetrics, University Medical Center Göttingen, Göttingen, Germany
5) Department of General, Visceral, and Pediatric Surgery, University Medical Center Göttingen, Germany
6) Institute for Pathology, Hannover Medical School, Hannover, Germany
7) Charité – Universitätsmedizin Berlin, Department of Hematology, Oncology, and Cancer Immunology, Berlin, Germany.
8) Department of Gastroenterology, Hepatology, Infectious Diseases and Endocrinology, Hannover Medical School, Hannover, Germany
9) Department of Pathology, Stony Brook University, Stony Brook, NY

* Corresponding author: ramona.schulz-heddergott@med.uni-goettingen.de

Figure 1D

HCT116 RKO

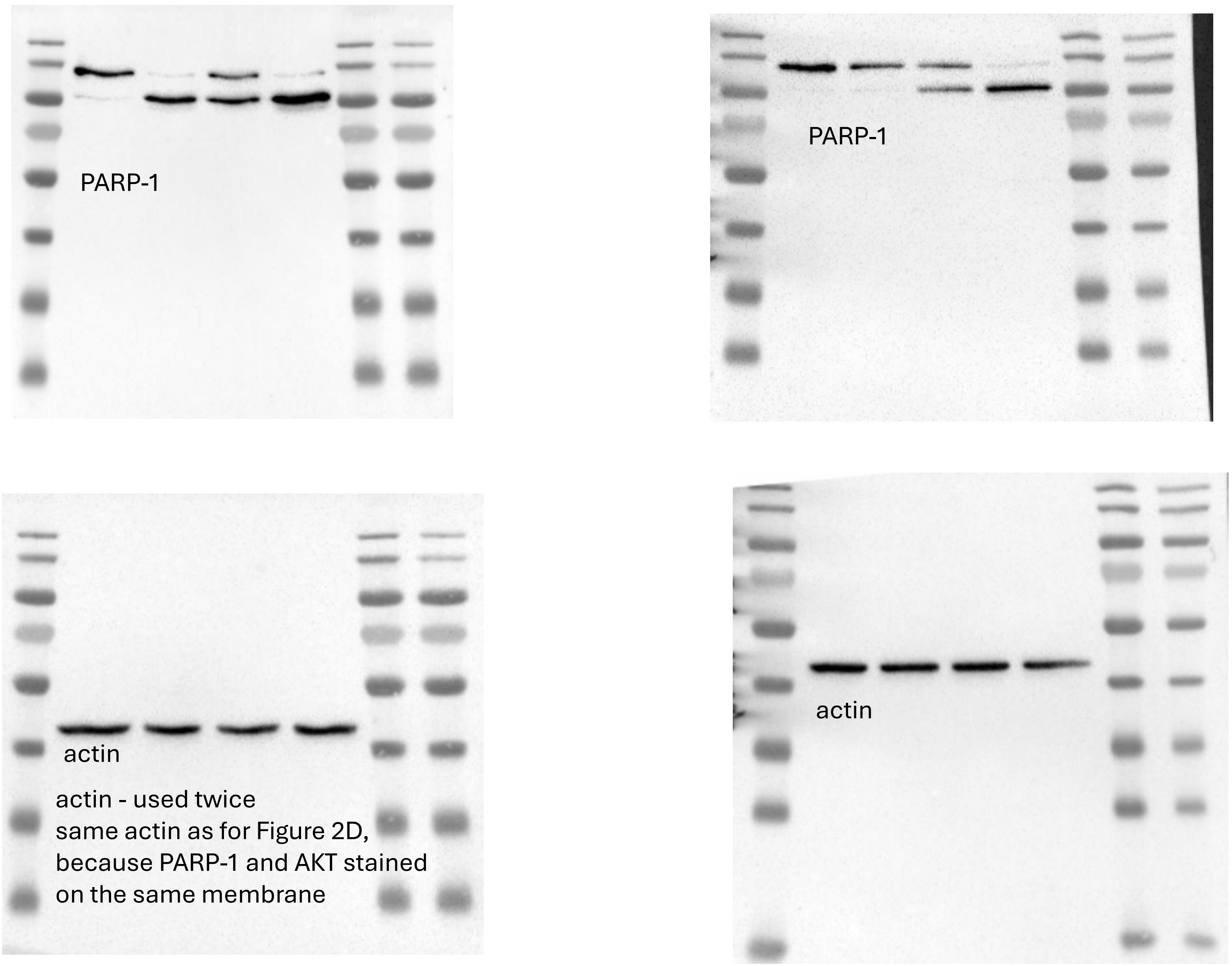

Figure 2C

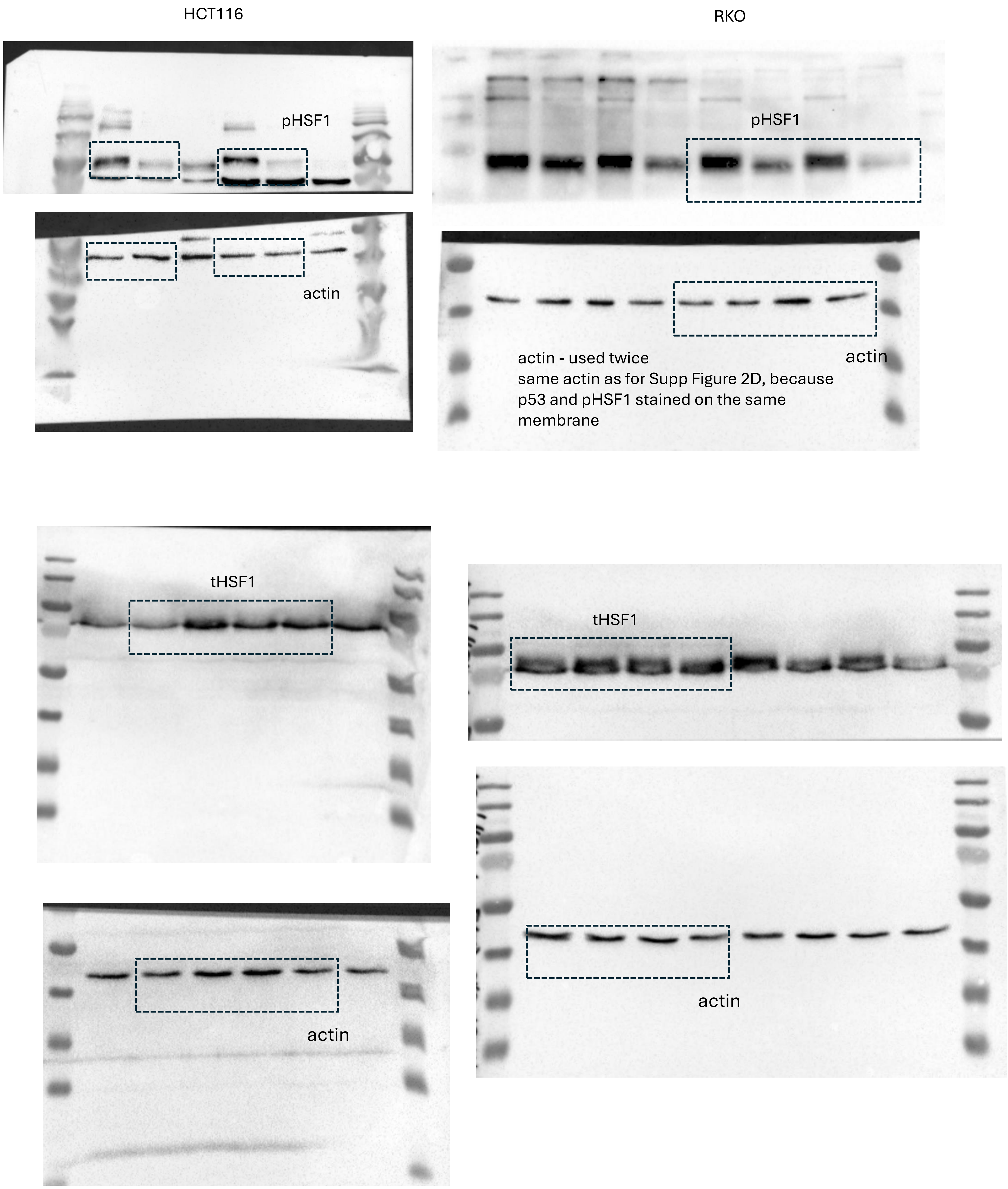
HCT116
RKO
pHSF1
pHSF1
actin
actin - used twice
same actin as for Supp Figure 2D, because
p53 and pHSF1 stained on the same
membrane
actin
tHSF1
tHSF1
actin
actin

Figure 2D

HCT116

RKO

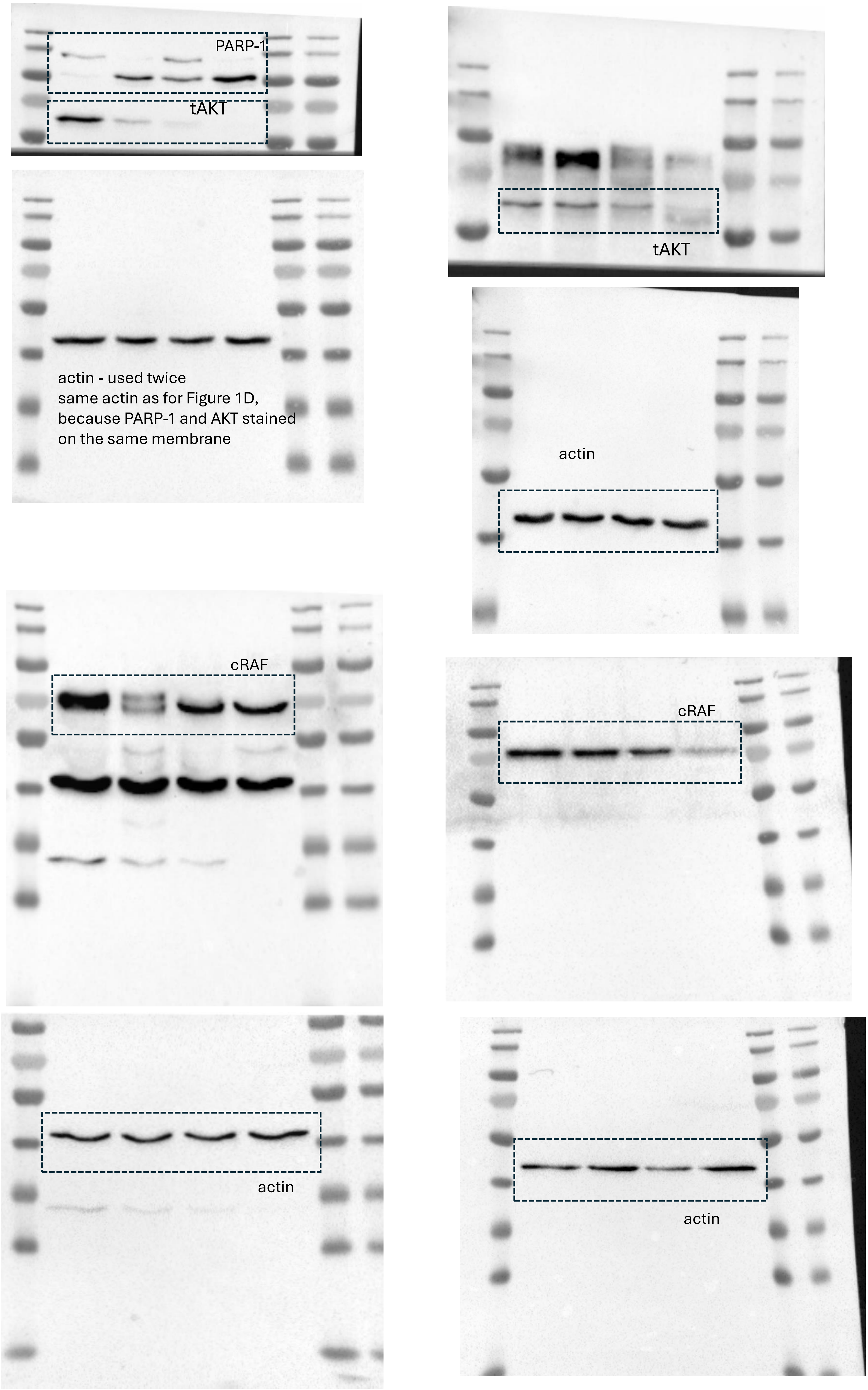

Figure 5

HCT116

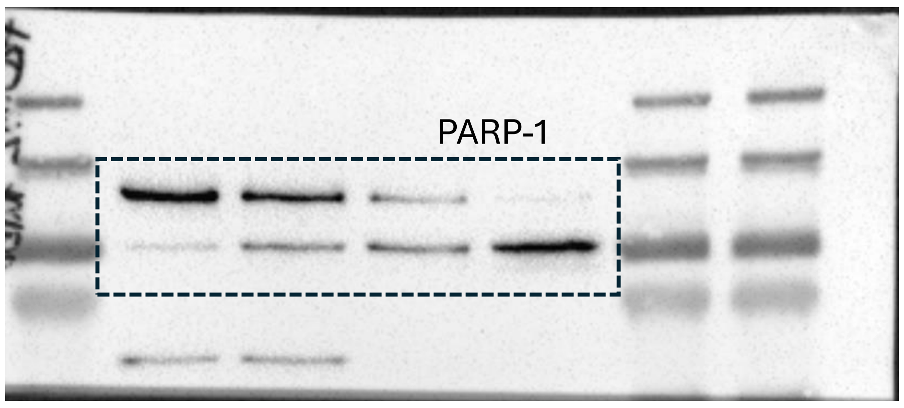

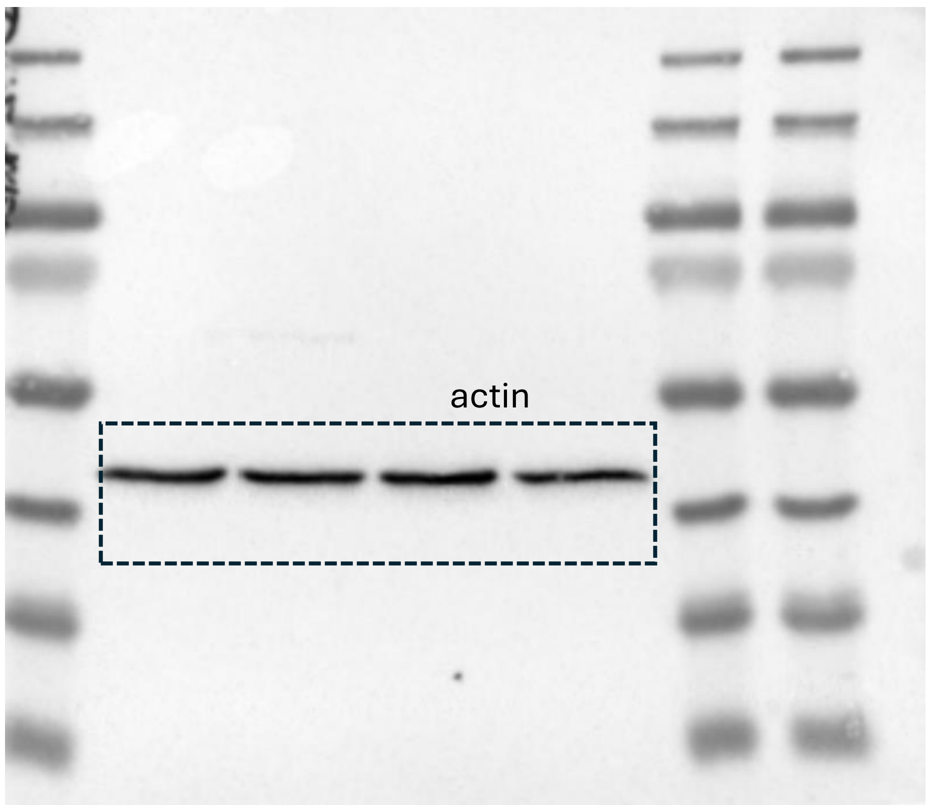

Figure 6B

HCT116

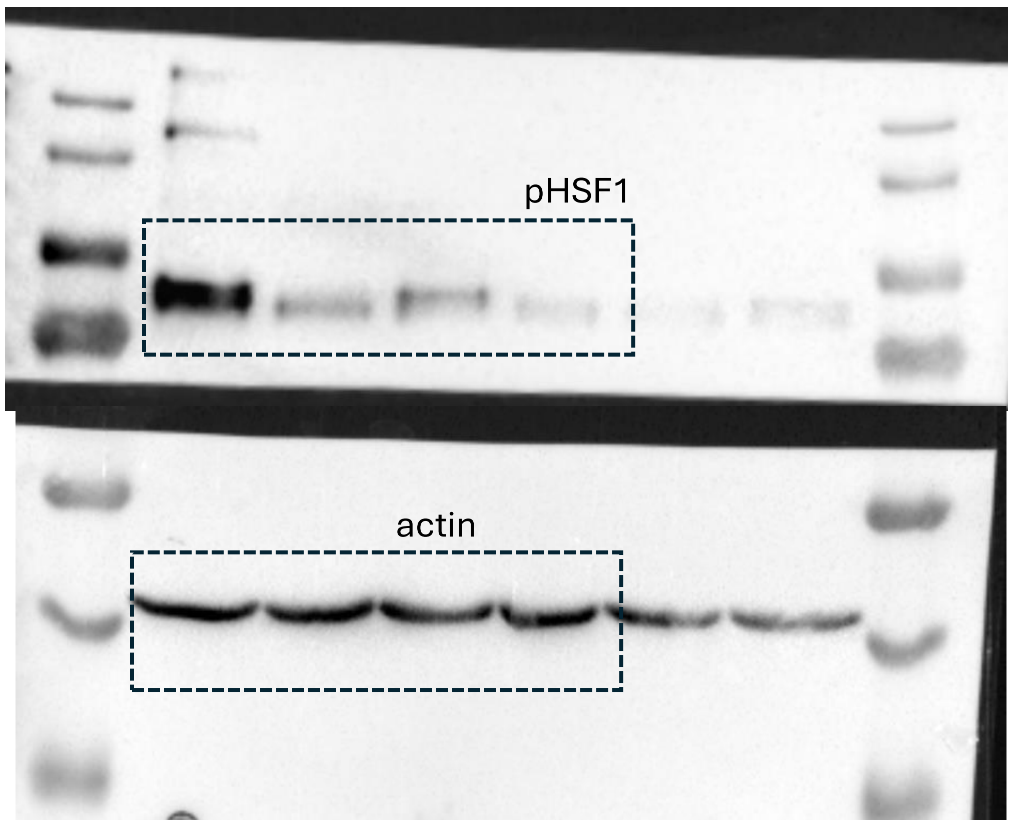

Figure 6C

HCT116

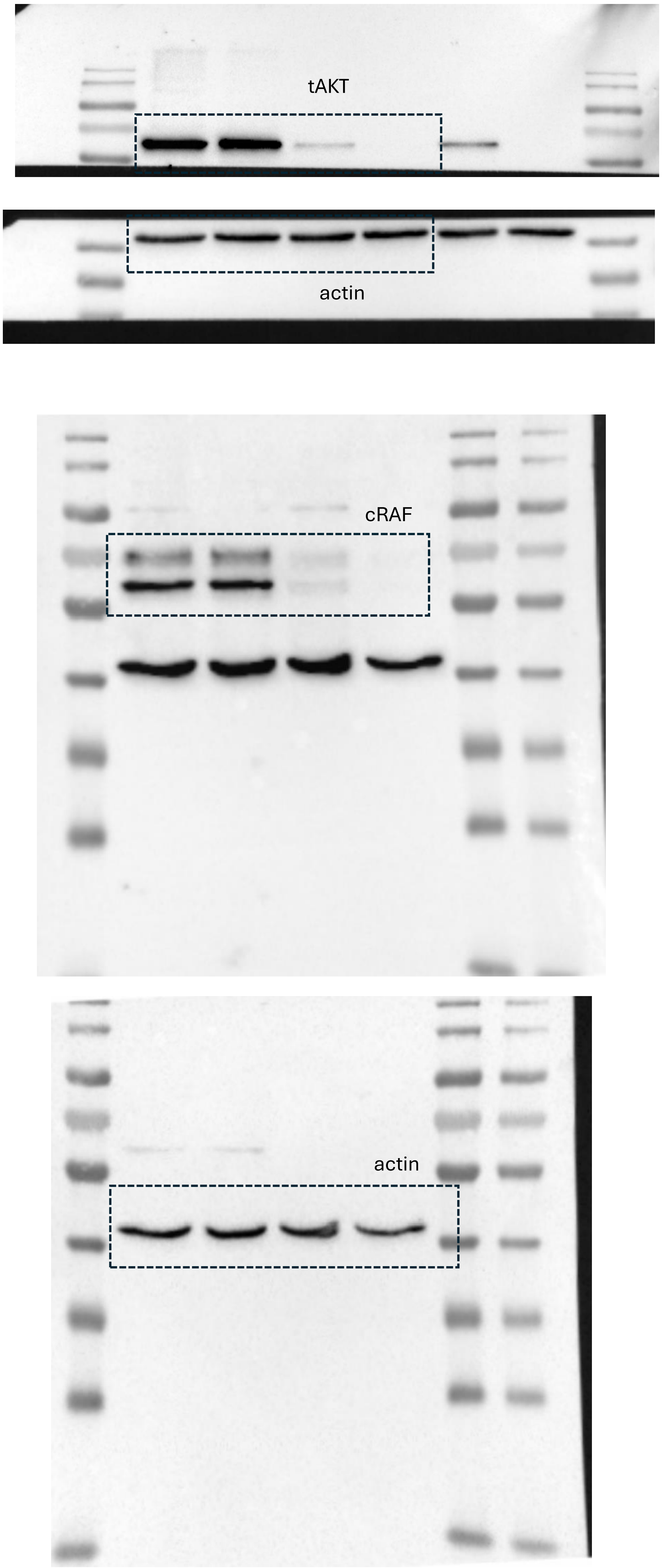

Figure 6F

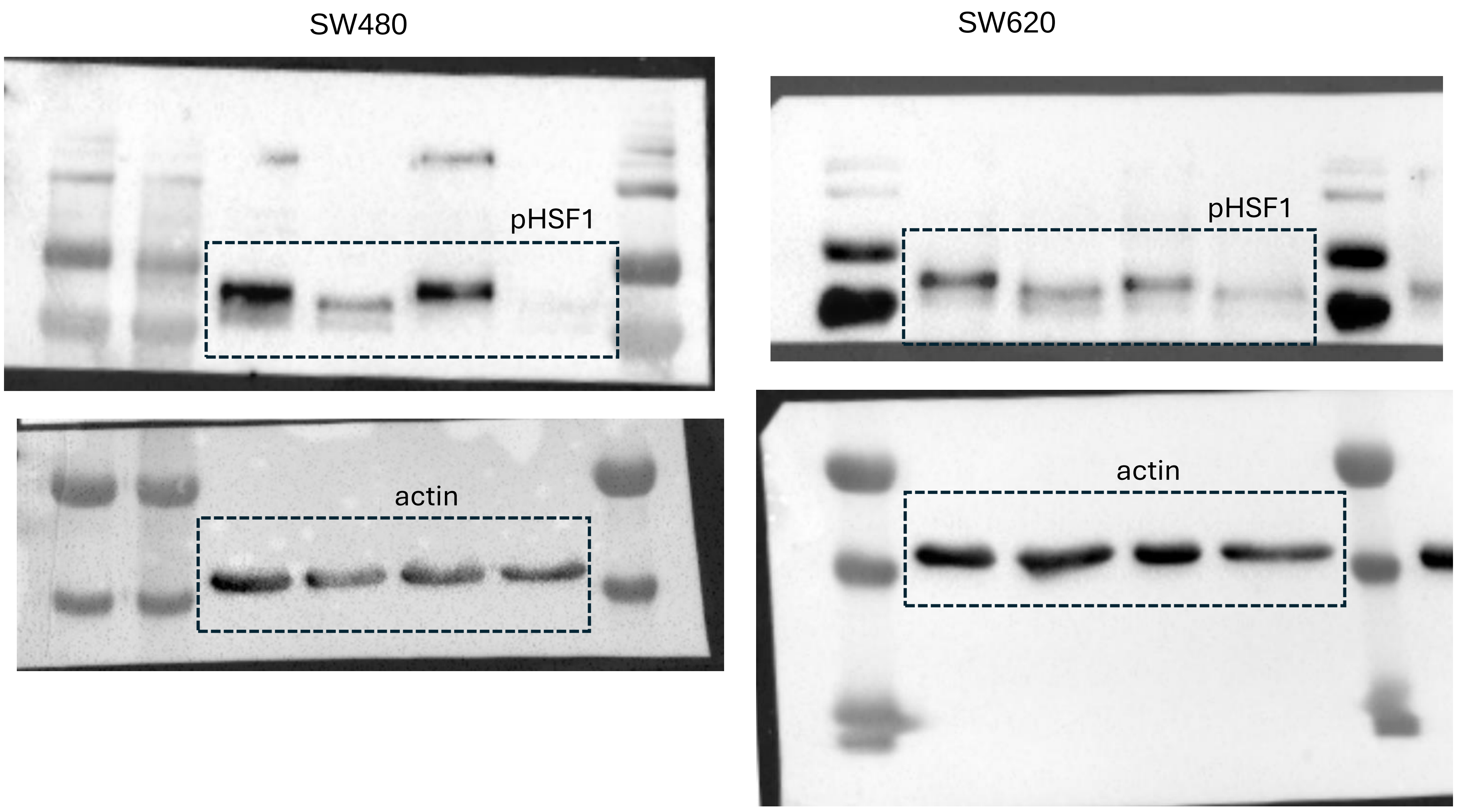

Supplementary Figure 2D

HCT116

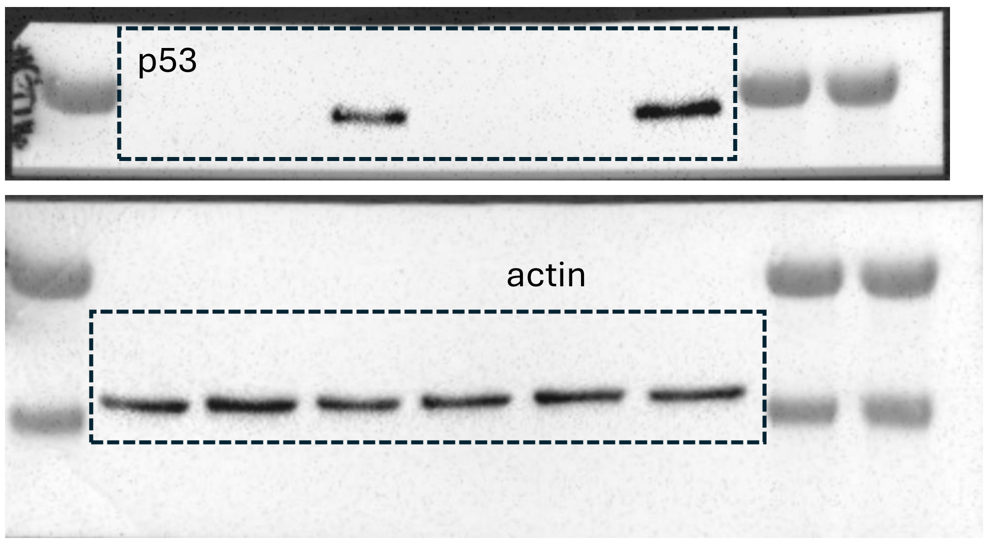

RKO

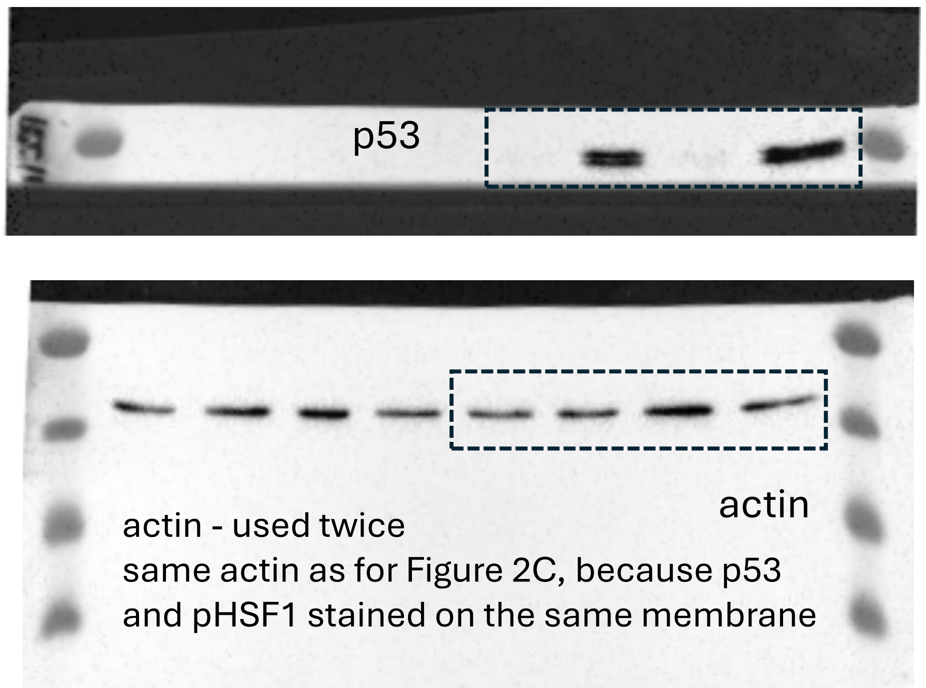

Supplementary Figure 5

RKO

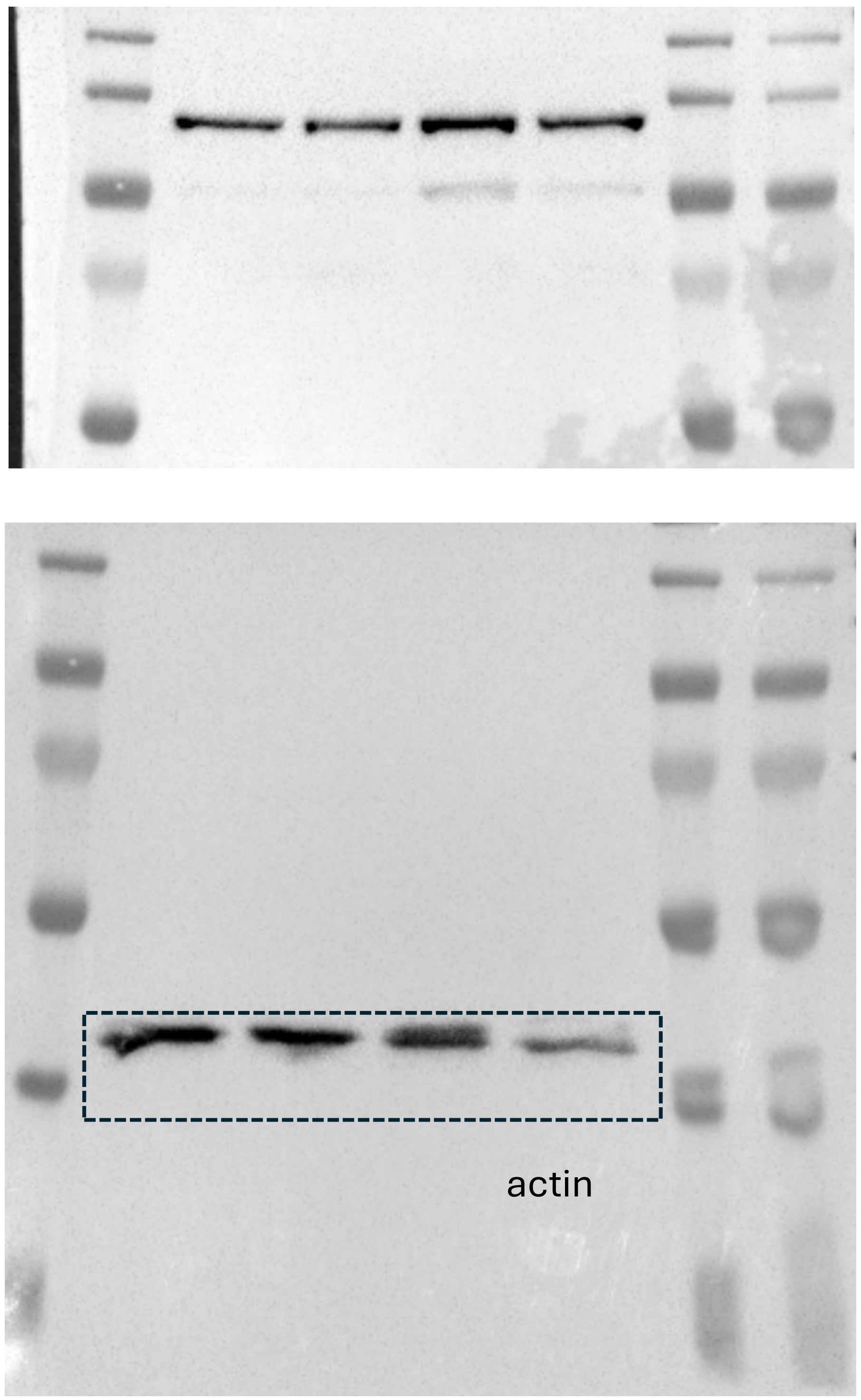

Supplement: Supplementary file 2 — Original Data Files [file 41418_2025_1502_MOESM2_ESM.pdf]
